# Residential Therapy With Navigated Transcranial Magnetic Stimulation for Combat-Related PTSD: A Randomized Clinical Trial

**DOI:** 10.1001/jamanetworkopen.2026.5110

**Published:** 2026-04-07

**Authors:** Peter T. Fox, Felipe S. Salinas, John D. Roache, Marlon Quinones, Phillip W. Vaughan, Crystal Franklin, Casey L. Straud, Larry Price, Mary Unzueta-Hernandez, Mary K. Woolsey, Angela M. Chavez, Deborah A. Hoselton, Antoinette R. Brundige, Brett T. Litz, Stacey Young-McCaughan, Terence M. Keane, Alan L. Peterson

**Affiliations:** 1 Research Imaging Institute, University of Texas Health Science Center at San Antonio (UTHSCSA); 2 Department of Radiology, University of Texas Health Science Center at San Antonio (UTHSCSA); 3 Department of Psychiatry and Behavioral Science, University of Texas Health Science Center at San Antonio (UTHSCSA); 4 Departments of Neurology and Pharmacology, University of Texas Health Science Center at San Antonio (UTHSCSA); 5 Research and Development Service, South Texas Veterans Health Care System, San Antonio; 6 iKare Mood Trauma Recovery Clinic, San Antonio, Texas; 7 FIRST-MD, San Antonio, Texas; 8 Texas Department of Information Resources, Austin; 9 Department of Psychology, University of Texas at San Antonio; 10 Mission Resiliency, Laurel Ridge Treatment Center, San Antonio, Texas; 11 Massachusetts Veterans Epidemiological Research and Information Center, Boston; 12 Behavioral Science Division, National Center for PTSD, VA Boston Healthcare System, Boston, Massachusetts; 13 Department of Psychiatry, Boston University School of Medicine, Boston, Massachusetts

## Abstract

**Importance:**

Improved treatments for combat-related posttraumatic stress disorder (PTSD) are needed.

**Objective:**

To determine whether navigated transcranial magnetic stimulation (TMS) is efficacious when added to intensive residential therapy for combat-related PTSD.

**Design, Setting, and Participants:**

A 1:1 randomized clinical trial with 30-day treatment and 1- and 3-month follow-ups was conducted in a residential facility specializing in military mental health with geographically broad catchment. Enrollment was from July 2017 to March 2019. Participants were military personnel with PTSD recruited from treatment facility admissions. Patients, staff, and assessors were blinded to treatment arm. Data were analyzed from November 2020 to April 2023.

**Interventions:**

Both trial arms received intensive residential treatment as standard of care (SoC). SoC included manualized prolonged exposure twice per week combined with daily, day-long psychotherapeutic augmentations as a 30-day program. Navigated TMS (active or sham) was added to SoC. TMS was individually targeted both anatomically (structural) and connectomically (functional) using magnetic resonance imaging and delivered by robotic stereotaxy. TMS was delivered daily (7 d/wk) for up to 20 sessions.

**Main Outcomes and Measures:**

The primary outcome was symptom-severity change in PTSD Checklist for *DSM-5* (PCL-5) scores between baseline and end-of-treatment assessments. Secondary outcomes were Clinician-Administered PTSD Scale for *DSM-5* (CAPS-5) and Patient Health Questionnaire–9 (PHQ-9) depression module scores across treatment and follow-up phases.

**Results:**

Among 129 military personnel and veterans screened, 119 with moderate (9 [8%]), severe (56 [47%]), or extreme (54 [45%]) PTSD were randomized (60 to active TMS and 59 to sham TMS), with 6 dropouts (5%) before end of treatment. Mean (SD) participant age was 37.6 (6.5) years; 107 (90%) were male. For both treatment arms, all measures confirmed significant symptom relief relative to baseline. At end of treatment, both PTSD measures showed superior symptom relief for active TMS: mean PCL-5 score difference, −5.94 (95% CI, −11.77 to −0.10; *P* = .02); mean CAPS-5 score difference, −6.03 (95% CI, −10.84 to −1.22; *P* = .008). In follow-up, 2 measures showed superior symptom relief durability for active TMS: PCL-5 (mean score difference, −12.30; 95% CI, −22.03 to −2.57; *P* = .008) and PHQ-9 (mean score difference, −3.45; 95% CI, −0.03 to −6.86; *P* = .03). Reliable change indices showed superior symptom relief durability for active vs sham TMS on the PCL-5 (85% [95% CI, 71%-98%] vs 59% [95% CI, 41%-78%] at 1 month; *P* = .03).

**Conclusions and Relevance:**

In this randomized clinical trial of combat-related PTSD, navigated TMS was efficacious when added to intensive residential treatment. This suggests that navigated TMS neuromodulation can effectively reduce PTSD symptoms.

**Trial Registration:**

ClinicalTrials.gov Identifier: NCT02853032

## Key Points

**Question:**

Does adding navigated transcranial magnetic stimulation (TMS) to an intensive residential program for combat-related posttraumatic stress disorder (PTSD) improve outcomes?

**Findings:**

In this randomized clinical trial enrolling 119 military personnel and veterans, active navigated TMS provided greater reduction of PTSD symptoms during treatment and longer remissions than sham when added to trauma-focused prolonged exposure and cognitive behavioral treatments in an intensive residential setting. Clinically significant reductions in PTSD symptoms were achieved by 85% of active TMS vs 59% of sham recipients at 1-month follow-up.

**Meaning:**

These findings suggest that navigated TMS can be an efficacious addition to behavioral therapy for combat-related PTSD.

## Teaser

This randomized clinical trial explores whether adding navigated transcranial magnetic stimulation to a standard-of-care intensive residential prolonged exposure program improves posttraumatic stress disorder (PTSD) symptoms among active-duty military personnel or veterans.

## Introduction

Posttraumatic stress disorder (PTSD) is a debilitating disorder afflicting 4% to 17% of nearly 3 million US military personnel who were deployed in Iraq and Afghanistan.^1,2,3^ The ongoing war in Ukraine has heightened international awareness of the impact of combat-related PTSD on military personnel and civilians and the need for more effective treatments.^4,5^ Pharmacotherapy is widely prescribed for PTSD but often is ineffective, has unacceptable adverse effects, or both.^6^ Trauma-focused cognitive behavioral therapies (CBTs), including prolonged exposure (PE), are effective^7^ but have high dropout rates when delivered weekly.^8^ Massed PE (multiple sessions per week) shortens treatment duration and improves retention.^9,10^ Reinforcing massed PE with trauma-focused CBT in an intensive, full-day outpatient program improves symptom-relief durability.^11^ In principle, an intensive residential program of massed PE reinforced with CBT should provide the highest level of care for PTSD.^12,13^ Moreover, the inpatient setting is well suited for adding daily therapeutic interventions such as transcranial magnetic stimulation (TMS). To date, no randomized clinical trial (RCT) to our knowledge has quantified symptom reduction from intensive residential therapy for combat-related PTSD or assessed the efficacy of TMS as an add-on to massed PE, CBT, or both.

TMS therapy is cleared by the US Food and Drug Administration (FDA) for major depressive disorder (MDD) and obsessive-compulsive disorder but not yet for PTSD. US Department of Veterans Affairs–Department of Defense Clinical Practice Guidelines expressed “a great deal of interest in TMS for the treatment of PTSD,”^14^ citing small-sample TMS RCTs with substantial PTSD-symptom remediation.^15,16,17^ The present trial readdressed this therapeutic goal with advanced TMS methods and a larger sample. TMS was tested in combination with massed PE and trauma-focused CBT as standard of care (SoC). For both SoC and TMS, symptom-network disruption was the hypothesized mechanism of action.^18,19^

TMS engages multiregional neural circuits by network connectivity.^20,21,22,23,24^ The clinical effects of TMS, therefore, are best attributed to alterations in network behavior.^25^ Connectivity between the dorsolateral prefrontal cortex (DLPFC) and subgenual cingulate cortex (SGC) has long been implicated in mood disorders.^25,26,27,28,29,30,31^ This is the motivation for DLPFC being the predominant stimulation zone for TMS therapy for mental disorders, including PTSD. In the current trial, TMS target selection within the DLPFC was informed by connectivity-based parcellation,^32^ meta-analytic modeling of DLPFC-SGC connectivity,^33^ and PTSD symptom correlation with SGC activity.^34^

Interindividual variability in cortical morphology, cytoarchitectural borders, and functional connectivity argue that TMS targeting should be personalized by means of per-patient structural and functional imaging.^30^ Trials of navigated TMS in MDD validate this targeting principle.^35,36^ The present trial computed personalized treatment plans incorporating magnetic resonance imaging (MRI)–derived models of cortical surface folding, cortical column orientation, and functional connectivity combined with electromagnetic field modeling.^37,38,39^ Imaging-informed treatment plans were delivered by robotic, stereotactic navigation.^33,40,41,42^

In this RCT, navigated TMS (active or sham) was added to an intensive residential PTSD treatment program as SoC. It was hypothesized that (1) SoC would result in significant reductions in PTSD symptoms in both treatment arms and (2) adding TMS would improve therapeutic outcomes. A logistic objective was to evaluate the feasibility of delivering robot-navigated TMS in a TMS-naive facility.

## Methods

This RCT was approved by the institutional review board of the University of Texas Health Science Center at San Antonio; all participants provided written informed consent. The study followed the Consolidated Standards of Reporting Trials (CONSORT) 2025 guideline for clinical trials and adhered to policies and practices of the South Texas Research Organizational Network Guiding Studies on Trauma and Resilience.^43^

### Design and Sample

**Figure 1.**
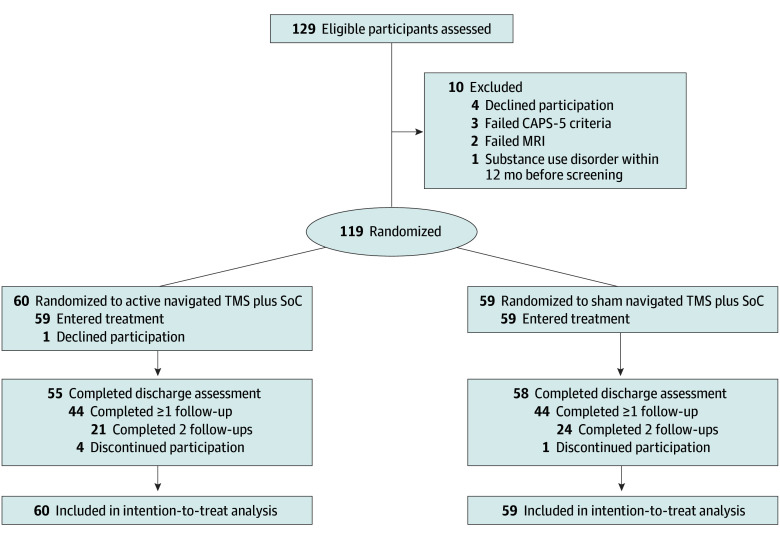
CONSORT Diagram Standard of care (SoC) was a residential therapy program. CAPS-5 indicates Clinician-Administered Posttraumatic Stress Disorder Scale for *DSM-5*; MRI, magnetic resonance imaging; TMS, transcranial magnetic stimulation.

Participants were recruited from 1 site: Military Medicine Service, Laurel Ridge Treatment Center (LRTC), San Antonio, Texas. Patient-referral sources for this cohort included 93 military bases in 26 states, Japan, and Germany. Enrollment was from July 2017 to March 2019. Study participation had 3 phases: lead-in, treatment, and follow-up. Lead-in included viewing a video depicting participant burden, establishing participant eligibility; baseline assessment; enrollment; MRI acquisition; and MRI-guided TMS-treatment planning. Eligible participants were randomized 1:1 by a central computer (Figure 1). During treatment, SoC and TMS were administered daily; symptom assessments and adverse events were obtained weekly. In follow-up, assessments were obtained online (self-report instruments) and by telephone (clinician-administered instruments) at 1 and 3 months posttreatment. No TMS booster sessions were possible after the treatment phase, as participants returned to duty stations (Supplement 1).

### Inclusion Criteria

Participants were active-duty or veteran military service members. Other inclusion criteria were age of 18 to 65 years; military deployment after September 11, 2001; admission to LRTC for treatment of combat-related PTSD; meeting Clinician-Administered PTSD Scale for *DSM-5* (CAPS-5)^44,45,46,47^ criteria for current (past month) PTSD; and a total PTSD Checklist for *DSM-5* (PCL-5) score of 25 or higher (score range, 0 [no symptoms] to 80)^47,48,49,50^ (Supplement 1).

### Treatment Conditions

All participants received the LRTC residential PTSD program. This was combined with either active or sham navigated TMS.

#### Residential Treatment Program

Individual, manualized PE was administered twice weekly for up to 6 sessions.^51,52^ PE included imaginal exposure, in vivo exposure, trauma processing, psychoeducation, and breathing retraining. Additionally, SoC incorporated daily, day-long activities-based PE augmentation^53,54^ (eAppendix 1 in Supplement 2).

#### Transcranial Magnetic Stimulation

TMS treatments were administered daily, 7 days per week, at a frequency of 20 Hz for 1600 pulses per session to a maximum of 20 sessions. TMS was not temporally coordinated with any specific SoC activity. TMS therapy was planned and delivered with a stereotactic navigation system using the cortical column cosine (C3) model for TMS targeting.^33,37,38,39,40,41,42^ The C3 model predicted TMS-induced functional brain responses, both locally^55,56,57^ and connectomically.^22,23,24,33^ Personalized treatments were created with treatment planning software applied to per-participant structural and functional MRI. Treatment plans targeted right anterior DLPFC in sites strongly connected with SGC.^32^ Connectivity of either sign (positive or negative) was permitted, as the TMS mechanism of action was conceptualized as disrupting mutually reinforcing interactions among nodes in a symptom network.^18,19^ Treatments were navigated by a robotic navigation system algorithmically similar to that previously reported.^39,40,41,42^ TMS planning and delivery were identical for both treatment arms; participants and all personnel were fully blinded (eAppendix 2 and eFigure 1 in Supplement 2).

### Measures

The primary outcome measure was change in PCL-5 score^47,48,49,50^ at the end of treatment vs baseline. Secondary outcome measures were change in CAPS-5 score^44,45,46,47^ (range, 0-80, with higher scores indicating greater symptom severity) and Patient Health Questionnaire–9 (PHQ-9) depression module score^57,58^ (range, 0-27, with higher scores indicating greater symptom severity). PCL-5 and PHQ-9 were obtained at 6 time points: baseline, weekly during treatment (weeks 1 and 2), end of treatment (week 3), and 1- and 3-month follow-ups. CAPS-5 was obtained at 3 time points: baseline and the 1- and 3-month follow-ups, with the 1-month follow-up functioning as end of treatment for statistical analyses.

Military service branch, rank, deployment information, marital status, and race and ethnicity were self-reported. Race and ethnicity are reported to convey generalizability; categories were African American, Hispanic, non-Hispanic White, and other (included Native American and Pacific Islander). Clinical status on admission was assessed by the Generalized Anxiety Disorder–7 questionnaire,^59^ Alcohol Use Disorder Identification Test,^60^ and Veterans RAND 12-Item Health Survey.^61^ Adverse events were assessed weekly during treatment and at each follow-up. A treatment-arm belief questionnaire was administered at end of treatment.

### Power Analysis

Sample size was determined for 80% power with 1-tailed 5%-level hypothesis tests designed to detect active TMS (plus SoC) improvements over sham TMS (plus SoC) across treatment phases in the primary outcome measure (PCL-5), assuming a treatment-effect size of 0.5 at end of treatment. Efficacy analyses were performed on the intention-to-treat sample of all randomized participants (Supplement 1).

### Statistical Analysis

Treatment-group differences for change over time in symptom scores were analyzed using linear growth models^62,63^ on the intention-to-treat sample. For each outcome, a piecewise approach was taken such that linear slope from baseline to end of treatment and from end of treatment to 3-month follow up were separately estimated. As applied, all available observations were used in growth-model estimations both per participant and armwise; missing data were not imputed. Effect sizes were computed with Hedges *g*. Reliable change indices (RCIs) were used to evaluate the proportion of participants who demonstrated reliable change at end of treatment and at the 1-month and 3-month follow-up.^64,65,66^ Two exploratory analyses were performed, testing whether uncontrolled variables were associated with the outcome. These were (1) valence (positive or negative) of functional connectivity between DLPFC and SGC and (2) treatment-arm belief in participants and evaluators (Supplement 1).

Data were analyzed from November 2020 to April 2023 using R, version 4.5.2 (R Project for Statistical Computing), and HLM 8 for Windows (Scientific Software International, Inc). One-sided *P* < .05 was considered significant.

## Results

### Participants

Of 129 active military personnel and veterans screened, 119 with moderate (9 [8%]), severe (56 [47%]), or extreme (54 [45%]) PTSD passed screening, completed baseline evaluations, and were randomized: 60 to active TMS and 59 to sham TMS (Figure 1). Mean (SD) participant age was 37.6 (6.5) years; 107 [90%] were men and 12 (10%) were women. A total of 25 (21%) were African American, 25 (21%) were Hispanic, 65 (55%) were non-Hispanic White, and 4 (3%) were other race and ethnicity. Demographic characteristics are reported in Table 1. Clinical characteristics are reported in eTable 1 in Supplement 2. Severe and extreme PTSD predominated in both treatment arms. Psychotropic medications on admission and changes during treatment are reported in eTable 2 in Supplement 2 and were similar in both arms. Six randomized participants (5%) discontinued participation prior to the week 1 assessment (5 [4%] active TMS, 1 [1%] sham). The remaining 113 (95%) received 15 or more TMS sessions in addition to their SoC treatment and completed discharge assessments. A full course of TMS (20 sessions) was completed by 50 of 58 sham-arm participants (86%) and 42 of 55 active-arm participants (76%).

**Table 1.** Demographic Characteristics of Enrolled Participants

Characteristic	Participants, No (%)^a^		
	Total (N = 119)	Sham TMS (n = 59)	Active TMS (n = 60)
Age, mean (SD) [range], y	37.6 (6.5) [21-55]	38.1 (6.1) [25-55]	37.1 (6.8) [21-52]
Sex			
Men	107 (90)	52 (88)	55 (92)
Women	12 (10)	7 (12)	5 (8)
Marital status			
Married	88 (74)	41 (69)	47 (78)
Single	12 (10)	6 (10)	6 (10)
Divorced	18 (15)	11 (19)	7 (12)
Widowed	1 (1)	1 (2)	0
Educational level			
High school	19 (16)	7 (12)	12 (20)
1-3 y College	78 (66)	37 (63)	41 (68)
≥4 y College	22 (18)	15 (25)	7 (12)
Race and ethnicity			
African American	25 (21)	12 (20)	13 (22)
Hispanic	25 (21)	10 (17)	15 (25)
Non-Hispanic White	65 (55)	35 (59)	30 (50)
Other^b^	4 (3)	2 (3)	2 (3)
Service status			
Active	114 (96)	58 (98)	56 (93)
Not active	5 (4)	1 (2)	4 (7)
Military rank^c^			
E-4 to E-6	49 (41)	30 (51)	19 (32)
E-7 to E-9	52 (44)	23 (39)	29 (48)
WO-1 to WO-5	18 (15)	7 (12)	11 (18)
Service branch			
Army	42 (35)	33 (56)	39 (65)
Air Force	26 (22)	16 (27)	10 (17)
Navy	11 (9)	4 (7)	7 (12)
Marines	10 (8)	6 (10)	4 (7)
Years of service, mean (SD) [range]	16.6 (6.0) [3-31]	16.9 (5.7) [5-30]	16.4 (6.2) [3-31]
Deployments, No.^d^			
1	15 (13)	9 (16)	6 (10)
2	24 (20)	10 (17)	14 (24)
3	33 (29)	18 (31)	15 (25)
≥4	45 (38)	21 (36)	24 (41)

Abbreviation: TMS, navigated transcranial magnetic stimulation.

^a^ Cell counts vary based on available data across variables.

^b^ Included Native American and Pacific Islander.

^c^ E-4 to E-6 indicates junior noncommissioned officer; E-7 to E-9, senior noncommissioned officer; and WO, warrant officer.

^d^ Deployment data were unavailable for 1 sham TMS participant and 1 active TMS participant.

### PTSD Symptom Changes

**Figure 2.**
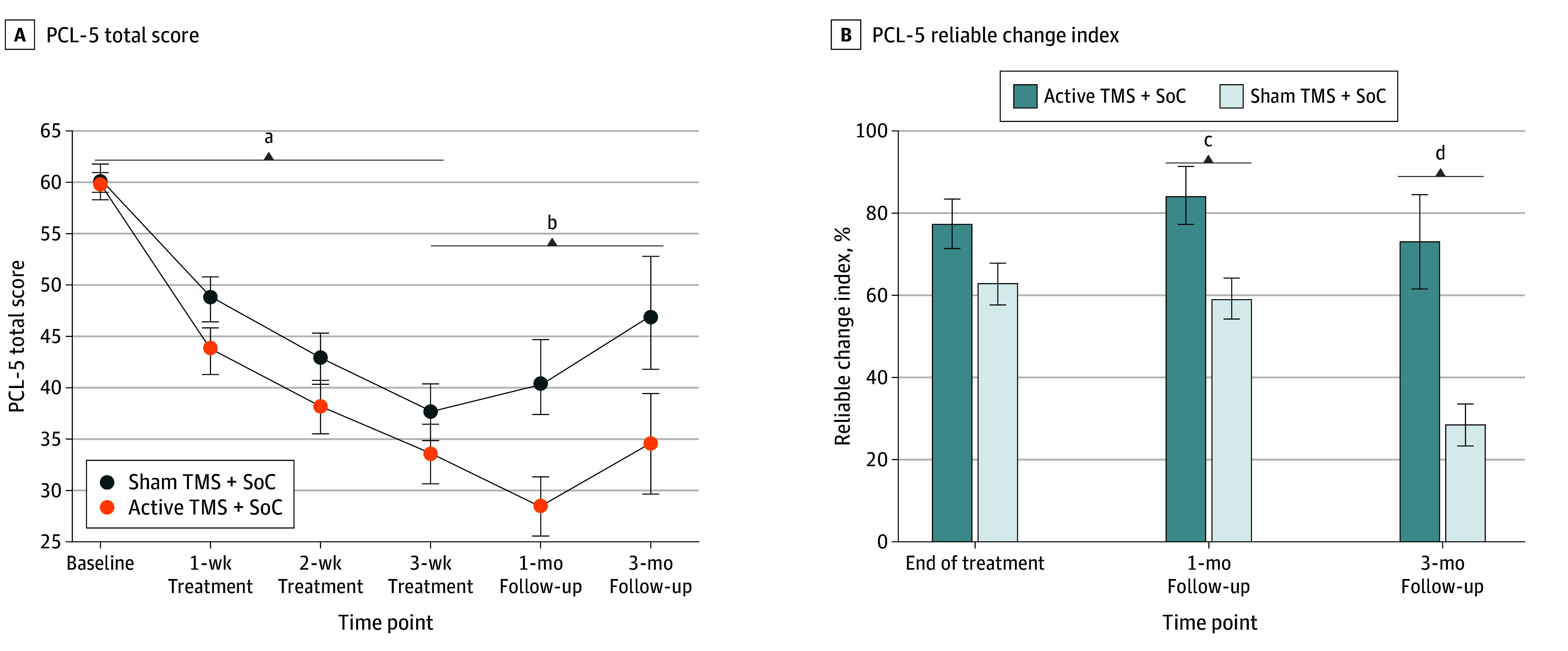
Line and Bar Graphs of Posttraumatic Stress Disorder Checklist for *DSM-5* (PCL-5) Outcomes by Treatment Group Error bars indicate plus or minus 1 SE. A, Change in PCL-5 score at the 3-week treatment (end of treatment) vs baseline was the primary outcome (PCL-5 scale range, 0 [no symptoms] to 80). B, Reliable change index scale ranges from 0% (no patients exhibited reliable change from baseline) to 100%. SoC indicates standard of care (residential therapy program); TMS, navigated transcranial magnetic stimulation. ^a^*P* < .001 for within-group treatment phase differences from baseline in both treatment arms and *P* < .05 for between-group differences by study phase. ^b^*P* < .01 for between-group differences by study phase. ^c^*P* < .05. ^d^*P* < .01.

Descriptive statistics for PTSD symptoms by time point are shown in Table 2. PCL-5 scores were available in sham vs active participants as follows: baseline, 59 (100%) vs 60 (100%); week 1, 58 (98%) vs 55 (92%); week 2, 58 (98%) vs 53 (88%); week 3 (end of treatment), 54 (92%) vs 49 (82%); 1-month follow-up, 27 (46%) vs 26 (43%); and 3-month follow-up, 14 (24%) vs 15 (25%). The mixed-effects models estimated mean-intercept differences between groups at occasions of interest (ie, end of treatment, 3-month follow-up), slopes for each group over time periods of interest (ie, baseline to end of treatment, end of treatment to 3-month follow-up), and between-group slope differences. Modeled between-group intercept differences by study phase are shown in Table 3. For PCL-5 scores, modeling from baseline to end of treatment (Figure 2A) revealed a significant decrease in symptoms (negative slopes) separately for both the active (*t*_325_ = −14.26; *P* < .001) and sham (*t*_325_ = −12.34; *P* < .001) groups. A significant group-by-time interaction for slope indicated that symptoms declined more for the active than sham group (*t*_325_ = −1.72; *P* = .04). The estimated mean PCL-5 score difference between the treatment groups at end of treatment was −5.94 (95% CI, −11.77 to −0.10; *t*_325_ = −2.00; *P* = .02) (Table 3). The greatest within-arm PTSD symptom reduction from baseline was in the active group at the 1-month follow-up (mean PCL-5 score difference, −31.22 [95% CI, −36.39 to −26.05]; Hedges *g*, −1.93). Between-arm modeling of the PCL-5 from end of treatment to 3-month follow-up revealed a significant return of symptoms within the sham group (*t*_78_ = 2.68; *P* = .009) but not the active group (*t*_78_ = 0.10; *P* = .92). A significant group-by-time interaction verified that PTSD symptoms for the active group differed from the sham group by remaining essentially constant over this period (*t*_78_ = −1.85; *P* = .03). The estimated mean PCL-5 score difference between the treatment groups at 3-month follow-up was −12.30 (95% CI, −22.03 to −2.57; *t*_325_ = −2.48; *P* = .008) (Table 3).

**Table 2.** PTSD Symptom Severity in Each Group by Time Point

Measure	Outcome						
	Baseline	End of treatment		1 mo Follow-up		3 mo Follow-up	
	Score, mean (SD) [Participants, No.]	Score, mean (SD) [Participants, No.]	Hedges *g*^a^	Score, mean (SD) [Participants, No.]	Hedges *g*^a^	Score, mean (SD) [Participants, No.]	Hedges *g*^a^
PCL-5							
Sham TMS + SoC^b^	60.24 (11.75) [n = 59]	37.65 (19.07) [n = 54]	−1.40	40.22 (22.81) [n = 27]	−1.07	46.64 (22.61) [n = 14]	−0.45
Active TMS + SoC^b^	59.57 (9.94) [n = 60]	33.49 (20.24) [n = 49]	−1.37	28.35 (14.57) [n = 26]	−1.93	34.47 (19.06) [n = 15]	−1.10
CAPS-5							
Sham TMS + SoC^b^	45.90 (8.25) [n = 59]	NA	NA	33.63 (11.56) [n = 32]	−1.58	30.00 (14.93) [n = 25]	−1.56
Active TMS + SoC^b^	44.92 (8.57) [n = 60]	NA	NA	27.31 (14.57) [n = 36]	−1.87	27.88 (15.92) [n = 24]	−1.64

Abbreviations: CAPS-5, Clinician-Administered PTSD Scale for *DSM-5*; NA, not applicable; PCL-5, PTSD Checklist for *DSM-5*; PTSD, posttraumatic stress disorder; SoC, standard of care; TMS, navigated transcranial magnetic stimulation.

^a^ Hedges *g* was calculated by comparing within-arm scores at the end point of interest with baseline within-arm scores.

^b^ SoC was residential treatment.

**Table 3.** Modeled Between-Group Differences in PTSD Symptom Severity by Study Phase

Measure	Treatment phase^a^				Follow-up phase			
	MD (95% CI)^b^	*t* Statistic	*P* value	Hedges *g*^c^	MD (95% CI)^b^	*t* Statistic	*P* value	Hedges *g*^c^
PCL-5	−5.94 (−11.77 to −0.10)	−2.00	.02	−0.21	−12.30 (−22.03 to −2.57)	−2.48	.008	−0.57
CAPS-5^d^	−6.03 (−10.84 to −1.22)	−2.48	.008	−0.47	−2.84 (−9.82 to 4.14)	−0.80	.21	−0.14

Abbreviations: CAPS-5, Clinician-Administered PTSD Scale for *DSM-5*; MD, mean difference; PCL-5, PTSD Checklist for *DSM-5*; PTSD, posttraumatic stress disorder.

^a^ Treatment phase was baseline to end of treatment.

^b^ Compares active with sham navigated transcranial magnetic stimulation by study phase. Modeled MD was calculated as the active group intercept minus the sham group intercept at conclusion of the phase. Negative mean difference indicates greater symptom remediation by active transcranial magnetic stimulation.

^c^ Hedges *g* was calculated between arms at the time point of interest based on descriptive statistics.

^d^ For CAPS-5, 1-month follow-up was used as the end of treatment for the treatment phase calculation.

CAPS-5 scores were available (sham vs active) as follows: baseline, 59 (100%) vs 60 (100%); 1-month follow-up, 32 (54%) vs 36 (60%); and 3-month follow-up, 25 (42%) vs 24 (40%). For CAPS-5 scores, growth modeling indicated significant decreases in symptoms (slopes) from baseline to 1-month follow-up for both the active (*t*_66_ = −9.35; *P* < .001) and sham (*t*_66_ = −6.32; *P* < .001) groups separately. An interaction effect indicated greater decreases for the active than the sham group (*t*_66_ = −1.88; *P* = .03). The modeled mean-intercept difference between the treatment groups at the 1-month follow-up was −6.03 (95% CI, −10.84 to −1.22; *t*_117_ = −2.46; *P* = .008) (Table 3). The greatest CAPS-5 reduction from baseline was in the active group at the 1-month follow-up (mean difference, −17.61 [95% CI, −21.82 to −13.40]; Hedges *g* = −1.17). Mixed-effects modeling of the CAPS-5 slopes from end of treatment to the 3-month follow-up revealed no significant changes within the sham (*t*_34_ = −1.15; *P* = .25) or active (*t*_34_ = 0.20; *P* = .84) groups and no interaction with time (*t*_34_ = 0.96; *P* = .34). The modeled mean-intercept difference between groups at the 3-month follow-up was −2.84 (95% CI, −9.82 to 4.14; *t*_79_ = −0.80; *P* = .21).

### PTSD Reliable Change Index

For PCL-5, 38 of 53 participants (72%) exceeded the RCI threshold (PCL-5 reduction of ≥15 points^66^) at the 1-month follow-up and 15 of 29 (52%) at the 3-month follow-up (Figure 2B). The proportion of participants exceeding the RCI threshold on the PCL-5 was greater in the active compared with the sham group at the 1-month follow-up (85% [95% CI, 71%-98%] vs 59% [95% CI, 41%-78%]; χ^2^_1_ = 4.61; *P* = .03) and the 3-month follow-up (73% [95% CI, 51%-96%] vs 29% [95% CI, 5%-52%]; χ^2^_1_ = 7.26; *P* = .007).

On the CAPS-5, 38 of 68 participants (56%) exceeded the RCI threshold (PCL-5 reduction of ≥12 points^66^) at the 1-month follow-up and 27 of 49 (55%) at the 3-month follow-up. The proportion of participants exceeding the RCI threshold on the CAPS-5 was not significantly different in the active (23 of 36 [64%]) compared with the sham (15 of 32 [47%]) group at the 1-month follow-up (χ^2^_1_ = 2.04; *P* = .15) or 3-month follow-up (active, 16 of 24 [67%]; sham, 11 of 25 [44%]; χ^2^_1_ = 2.69; *P* = .10).

### MDD Symptom Changes

Both treatment groups exhibited progressive, moderate decreases in depressive symptom (PHQ-9) severity at end of treatment (eFigure 2 in Supplement 2). Descriptive statistics and key pairwise comparisons across outcomes are presented in eTable 4 in Supplement 2. Growth modeling of PHQ-9 scores (eTable 5 in Supplement 2) showed that both the active (*t*_325_ = −10.28; *P* < .001) and sham (*t*_325_ = −10.97; *P* = .001) groups demonstrated significant linear decreases from baseline to end of treatment with no significant interaction (*t*_325_ = 0.20; *P* = .84), suggesting that depressive symptom reduction did not differ between groups. In follow-up, superior symptom relief durability was shown for active TMS (PHQ-9 mean score difference, −3.45; 95% CI, −0.03 to −6.86; *P* = .03).

### Exploratory Analyses

Valence of functional connectivity (ie, positively or negatively correlated) between DLPFC and SGC was positive in 47 participants (39%) and negative in 61 (51%); data were unavailable for 11 participants (9%). Connectivity valence (positive vs negative) was not associated with any outcome measure in either treatment arm or in either study phase. Treatment-arm belief was available for 108 participants (91%). Treatment-arm belief was not significantly associated with any outcome measure, either alone or in interaction with treatment group in either study phase.

### Adverse Events

Adverse events are reported in eTable 3 in Supplement 2. Headache was the most common adverse event, with similar frequency in both arms. Muscle contractions were more common in the active group. There were no serious adverse events.

## Discussion

This RCT in US military service members with PTSD (predominantly severe to extreme) post-9/11 found that navigated TMS provided before-to-after treatment improvements beyond those seen in the SoC intensive inpatient program. SoC (assessed by sham group outcomes) resulted in significant and meaningful reductions in self-reported and clinician-rated PTSD symptoms, comparable to those reported by recent RCTs of massed PE outpatient programs.^9,10,11^ Active TMS significantly added to the treatment gains of SoC. To our knowledge this was the first RCT of TMS used in combination with residential therapy of PTSD and the first to apply navigated TMS for PTSD. Robotic stereotactic navigation was also a logistic success, being reliably implemented in a TMS-naive facility. Efficacious treatments such as those evaluated herein are sorely needed to remediate the long-term consequences of PTSD in military service members, veterans, and civilians.

TMS dosing parameters are relevant when comparing this study with prior TMS trials. In this study, 20 Hz TMS was delivered daily (1600 pulses) to a 20-session maximum (32 000 total pulses) at approximately 105% MT. These lower-than-typical session numbers (20 vs 40) and the omission of booster sessions were constraints imposed by the limited duration (30 days) of the SoC program. The higher-than-typical stimulation rate (20 vs 10) increased total pulses delivered, offsetting the lower session number. Lower-than-typical intensity (approximately 105% vs 125% MT) emulated an effective prior trail^16^ to facilitate blinding and with the expectation that navigation would achieve an effective electric-field vector at a lower absolute E. As hypothesized, navigated TMS improved outcomes beyond SoC alone. By comparison, another TMS RCT^67^ delivered 2- to 4-fold more pulses over 20 to 30 sessions at a higher stimulus intensity (120% MT) but with conventional targeting; despite greater intensity and pulse number, no treatment benefits were found for PTSD or MDD symptoms. Notably, that study and the present RCT used identical TMS power supplies, coils, and blinding procedures and had comparable numbers of participants. Delivering TMS more often than daily (termed “accelerated TMS”^34,35^) has been cleared by the FDA for MDD and should further enhance the treatment gains observed herein from daily TMS.

In contrast to prevailing theory^25,28,30^ and a recent RCT of navigated TMS in MDD,^35,36^ the present trial targeted regions with strong DLPFC-to-SGC functional connectivity but did not select for negative (anticorrelated) functional connectivity. Rather, connectivity of either sign was allowed. Secondary analyses confirmed that connectivity sign had no influence on therapeutic outcomes. From this, we infer that TMS’s therapeutic mechanism of action might not be Hebbian (upregulating or downregulating the targeted pathway) or pacing (frequency entrainment). Rather, it suggests that TMS disrupts maladaptive, symptom-associated, reentrant neuronal firing patterns, allowing reestablishment of physiologic rhythms. By analogy to cardiac electromodulation, TMS defibrillates rather than paces. Broadly speaking, this mechanism of action is similar to that of trauma-focused behavior therapies, which seek to disrupt autonomic and cognitive reflexes to environmental cues.

### Limitations

This study has several limitations. Navigated TMS was added to an effective SoC: massed PE with CBT reinforcement. This design precludes inferences regarding TMS efficacy as monotherapy or when added to other PTSD therapies. Another limitation was that TMS was administered to a maximum of 20 daily sessions, fewer than typical TMS trials. TMS sessions were not time-locked to PE or CBT sessions, precluding inferences regarding optimal session timing. Additional research should address (1) whether multiple daily TMS sessions (accelerated TMS) enhance outcome, (2) whether more days of TMS give incremental benefit, and (3) interaction effects of time-locking TMS with PE or CBT sessions. Although network theory,^18,19^ preclinical studies,^20,21,22,23,24^ and between-study comparisons^31^ suggest that navigated TMS targeting and stereotactic delivery are more effective than conventional, nonnavigated targeting and hand delivery, the present trial was not a head-to-head comparison of navigated vs nonnavigated TMS.

In the patient sample obtained at a residential facility specializing in military medicine, severe and extreme PTSD predominated. This may limit applicability of results to less severe PTSD and nonmilitary populations. Although the residential setting minimized dropout during the treatment phase, during the follow-up phase, participant availability for assessment waned, limiting the reliability of treatment durability inferences. For CAPS-5 analysis, use of 1-month follow-up as a proxy for end of treatment was a limitation.

## Conclusions

In this RCT, navigated TMS added to residential SoC resulted in statistically significant and clinically meaningful improvements in PTSD symptoms that persisted during the 1- to 3-month follow-up for most participants. This study provides important new evidence of the therapeutic efficacy of navigated TMS combined with conventional therapy modalities. Before-to-after contrasts in the sham group also suggest the effectiveness of massed PE in residential settings is similar to that demonstrated previously in outpatient settings.^11^

## Notes

Supplement 1.
Trial Protocol

Supplement 2. **eAppendix 1.** Standard-of-Care Therapy **eAppendix 2.** Navigated TMS Methods **eFigure 1.** Robotic Stereotactic TMS Navigation System **eFigure 2.** PHQ-9 Outcomes by Treatment Group **eTable 1.** Participant Clinical Status on Study Entry **eTable 2.** Psychotropic Medications Alterations During Admission **eTable 3.** Adverse Events **eTable 4.** MDD Symptom Severity: Outcomes per Group by Time Point **eTable 5.** MDD Symptom Severity: Modeled Between-Group Differences by Study Phase

Supplement 3.
Data Sharing Statement
