# Supplementary material for: Residential Therapy With Navigated Transcranial Magnetic Stimulation for Combat-Related PTSD: A Randomized Clinical Trial
Source: JAMA Netw Open. 2026 Apr 7;9(4):e265110. doi: 10.1001/jamanetworkopen.2026.5110 (PMC13058764; doi:10.1001/jamanetworkopen.2026.5110)
Supplement: Supplement 1. — Trial Protocol [file jamanetwopen-e265110-s001.pdf]

SUPPLEMENT 1

CLINICAL TRIAL PROTOCOL & STATISTICAL ANALYSIS PLAN

Residential Therapy with Navigated TMS for Combat-Related Posttraumatic Stress Disorder: A Randomized Clinical Trial.

Peter T. Fox, MD, Felipe S. Salinas, PhD, John D. Roache, PhD, Jack L. Lancaster, PhD, Marlon Quinones, MD, Phillip W. Vaughan, PhD, Crystal Franklin, BS, Casey L. Straud, PsyD, Larry Price, PhD, Mary Unzueta-Hernandez, MD, Mary K. Woolsey, MS, Angela M. Chavez, LCSW, Deborah A. Hoselton, LPC, Antoinette R. Brundige, MA, Brett T. Litz, PhD, Stacey Young-McCaughan, PhD, Terence M. Keane PhD, Alan L. Peterson, PhD, for the Consortium to Alleviate PTSD

CONTENTS

IRB-approved Protocol: pages 1-16

Statistical Analysis Plan: pages 17-18

Protocol Template Form

**Item 1 UTHSCSA Tracking Number**	HSC20160191H

**Item 2 Abstract / Project Summary**	Provide a succinct and accurate description of the proposed research. State the purpose/aims. Describe concisely the research design and methods for achieving the stated goals. This section should be understandable to all members of the IRB, scientific and non-scientific.<br>**DO NOT EXCEED THE SPACE PROVIDED**.

Purpose/Objectives: Mounting amounts of evidence suggests that non-invasive stimulation of the dorsolateral prefrontal cortex (DLPFC) using image-guided, robotically-delivered transcranial magnetic stimulation (irTMS) maybe a safe and effective treatment modality for Posttraumatic Stress Disorder (PTSD). However the large variability in the magnitude of clinical outcomes reported is likely related to the current lack of knowledge of ideal location of stimulation and the limited precision in the targeting of brain circuits needed to obtain an optimal treatment response. In this protocol we will: 1) generate individualized treatment plans based on an individual's functional Magnetic Resonance Imaging (fMRI) and meta-analytical based connectivity analysis to guide the delivery of adjunct, irTMS to active duty military service members and veterans with PTSD participating in an intensive program providing integrated evidence-based psychotherapy and pharmacological management (Treatment as Usual (TAU)). 2) To use clinician ratings and self-report PTSD symptom scales, as well as other indicators of clinical change, to determine whether compared with TAU, addition of adjunct irTMS improves clinical outcomes. 3) To conduct neuroimaging-based assessments aimed to measure irTMS effects on network connectivity in service members and veterans receiving treatment for PTSD and the potential correlation of connectivity changes with clinical outcomes.

Research Design/Plan: Randomized, double-blind, sham-controlled clinical trial.

Methods: Consented participants receiving TAU for PTSD at LRTC will be randomized to receive 20 consecutive days of adjunct irTMS according to one of these two treatment arms: **Arm 1** TAU plus irTMS to the right DLPFC or **Arm 2** TAU plus sham irTMS to the right DLPFC. At UTHSCSA's Research Imaging Institute (RII), where all brain imaging and motor threshold assessments will be conducted, irTMS treatment plans will be generated based on (pre-treatment) anatomical and functional magnetic resonance imaging (fMRI) to guide the optimal robotic positioning of the TMS coil to accurately target each subject DLPFC. Participants will be assessed at baseline, during treatment, immediately following treatment and at 1-month post-treatment. A comparison of baseline brain connectivity measurements with subjects' fMRI conducted immediately following treatment will be conducted to identify network connectivity changes potentially associated to treatment response.

Clinical Relevance: This will be the first study to determine the clinical effects of adjunct irTMS delivered to the right DLPFC in the treatment of PTSD, to provide clues about the ideal neural networks to target for more robust clinical outcomes, and to identify potential biomarkers of treatment response including changes in brain networks connectivity.

**Item 3** Background	

Describe past experimental and/or clinical findings leading to the formulation of your study.<br>*For research involving unapproved drugs, describe animal and human studies.*<br>*For research that involves approved drugs or devices, describe the FDA approved uses of this drug/device in relation to your protocol.*	PTSD is a debilitating anxiety disorder experienced by over 7.8% of the United States populace over the course of their lifetime. Since 2001, over 2.5 million U.S. Service Members have been deployed in combat operations, resulting in higher rates of PTSD in combat veterans returning from Operation Iraqi Freedom (12-20%) and Operation Enduring Freedom (6-12%). Excellent recent reviews of TMS literature suggest that despite that pharmacologic or psychotherapeutic (e.g., evidenced based forms of cognitive behavioral therapy-CBT known as Prolonged Exposure-PE; or Cognitive Processing Therapy-CPT) and combination treatments have been shown to help reduce PTSD symptoms, there is no definitive treatment for PTSD. Beyond the limited success of currently available treatments in fully alleviating PTSD treatments, pharmacological approaches are also associated with frequent medication side effects leading to treatment discontinuation. Thus, there is an impetus to identify other "non-invasive" treatments—e.g. brain stimulation to reduce symptom burden in patients with PTSD. TMS (aka irTMS) is a non-invasive neuromodulatory treatment modality that is approved by regulatory bodies in the United States by the Food and Drug Administration (FDA), Canada, Europe and Australia for the treatment of Major Depressive Disorder (MDD) in patients failing to respond to at least one treatment trial. FDA approval for TMS treatment of MDD was based on a double-blind, placebo-controlled (sham TMS), multi-center clinical trial. In this trial, the neuroanatomical location targeted was the left DLPFC, based on functional neuroimaging evidence that its serves as a hub in a multi-node network dysregulated in MDD. A similar approach was adopted in previous TMS treatment trials of PTSD when targeting the right DLPFC. However, these TMS targeting methods are neurophysiologically, neuroanatomically and technically unsophisticated. Specifically, (left or right) DLPFC was located by reference to the hand region of primary motor cortex, rather than being based on per-subject anatomy or connectivity. The orientation in which the coil was positioned (with the E-field vector pointed toward the patient's nose) was (grossly) optimized for motor cortex but did not account for differences in gyral anatomy between locations or subjects; these studies were also not image-guided. This is the standard-of-care (SOC) TMS treatment of MDD. Furthermore, there is also a lack of strong evidence determining whether clinical outcomes differ when between TMS targeting of the right DLPFC and therefore modulation of different network connectivity. In the proposed trial, the treatment efficacy of TMS for the treatment of PTSD will be tested using a fundamentally new strategy to addresses the limitations of current TMS targeting methods and to identify suspected in differences in clinical outcomes linked to treating the right DLPFC.

**Item 4**<br>Purpose and rationale<br>*Insert purpose, objectives and research questions/hypotheses here.*<br>*If you cut and paste from another document, make sure the excerpted material answers the question*	We propose a randomized, double-blind, sham-controlled, 20 consecutive day trial of adjunct TMS to the right DLPFC in active duty service members and veterans with PTSD receiving an integrated and evidence-based treatment (i.e. TAU) for PTSD incorporating both adequate pharmacological approaches and Cognitive behavioral therapy (CBT) at Laurel Ridge Treatment Center (LRTC). In addition to TAU, consented patients will be randomized to receive irTMS in one of two treatment arms: <u>Arm 1</u> delivers active irTMS to the right DLPFC. <u>Arm 2</u> delivers sham TMS (inactive coil) to the right DLPFC. To ensure equivalent placebo effects across arms, sham TMS delivers the exact sound produced during active irTMS, therefore patients are unable to discriminate between sham and active TMS. Moreover, clinicians providing TAU at LRTC and researchers conducting clinical outcomes assessments will be blinded to each participants' treatment arm. Resting-state fMRIs (rs-fMRIs) will be used both for treatment planning (baseline imaging), which guides the individualized positioning of the TMS coil, and to characterize TMS-induced network connectivity (imaging treatment week 3). Self-report scales will be done weekly at LRTC. A 1-month post-treatment clinical follow-up will be conducted in person or over the phone to evaluate maintenance of clinical outcomes. Considering that depressive symptomatology, often meeting diagnostic criteria Major Depressive Disorder (MDD), is commonly observed in patients with PTSD, patients with comorbid PTSD and MDD won't be excluded from the study and we will also assess depressive symptoms throughout treatment at the same time intervals—i.e., baseline, weekly, and 1 month post-treatment.<br><br>***Aim 1. Test whether compared with TAU plus sham irTMS, TAU plus adjunct active irTMS is associated with greater clinical improvement that differs in specificity to PTSD or depressive symptomatology according to the targeting of right DLPFC .***<br>• *Hypothesis 1.* TAU plus adjunct active irTMS to the right DLPFC will be superior to TAU plus sham irTMS in relieving <u>PTSD symptoms</u>.<br><br>***Aim 2. Test for irTMS-induced changes in network functionality (plasticity) using rs-fMRI.***<br>• *Hypothesis 2a.* Intrinsic connectivity will be attenuated by active irTMS in networks connected to the respective treatment sites, as tested by correlation with the number of sessions received at the time of scanning and by pre-treatment-to-post-treatment contrast.<br>• *Hypothesis 2b.* Treatment-induced decreases in connectivity will correlate with symptom improvement within and across treatment arms.

**Item 5**<br>Study Population(s) Being Recruited<br><br>In your recruitment plan, how many different populations of prospective subjects do you plan to target? Provide number: 1	Identify the criteria for **inclusion**:	Identify the criteria for **exclusion**:

e.g., a population can be individuals with type 2 diabetes controlled with diet and/or a population of healthy controls. Or a population can be individuals attending an education program, etc.<br><br>List each different population on a separate row and provide a short descriptive **label**:<br>*(e.g., normal-healthy, diabetics, parents, children, etc.)*<br><br>*To add rows use copy & paste*		
Patients diagnosed by the LRTC with PTSD, receiving evidenced based combined pharmacological and psychotherapy (either PE or CPT)	**1)** Male or female English-speaking active duty or recently retired veteran patients who have deployed post 9/11 receiving treatment at LRTC between the ages of 18-65 years;<br>**2)** Patients must have a diagnosis of PTSD confirmed by the Clinician-Administered PTSD Scale (CAPS-5) at screening,<br>**3)** Subjects must have a minimum PTSD Symptom Checklist for DSM-V (PCL-5) symptom severity rating of 25.	**1)** Subjects with a diagnostic history of bipolar disorder, schizophrenia or schizoaffective disorder as documented in the medical record.<br>**2)** Substance use disorder during the 12 months prior to screening; except that Mild - Moderate, but not Severe, Alcohol Use Disorder (using DSM-5 criteria) will be allowed as determined by LRTC medical provider review.<br>**3)** Any history or signs of serious medical or neurological illness including seizure disorders. Except for seizures, a subject with a clinical abnormality may be included only if the study clinician considers the illness will not introduce additional risk and will not interfere with the study procedures. This will be determined during the screening phase via self-report and/or medical history review.<br>**4)** History of traumatic brain injury (TBI) with loss of consciousness for 20 minutes or more as determined by the History of Head Injuries questionnaire.<br>**5)** Females will be excluded if they are pregnant (i.e. positive pregnancy test identified after their LRTC intake).<br>**6)** Any history or signs of metal objects deemed unsafe for MRI or that may adversely affect image quality of the brain region (e.g. surgical clips, cardiac pacemakers, metal implants, etc.) in the body at the time of screening as indicated by self-report. MRI can have risks for persons with foreign bodies implanted in their body.

Item 6
Research Plan / Description of the Research Methods ***a.*** *Provide a **comprehensive narrative** describing the **research methods**.*
*Provide the **plan for data analysis** (include as applicable the **sample size calculation**).*

Step-by-Step Methods: We propose to test 100 patients (i.e. 50 per arm) who meet criteria for PTSD as determined by the Clinician Administered PTSD Scale (CAPS-5) and the outlined study inclusion criteria. We anticipate consenting and screening approximately 200 male and female subjects, in order to meet the target enrollment goals. To account for subject dropouts and retain a sample of 100 subjects completing the double-blind treatment phase (50 per treatment group), 132 eligible subjects (66 in each group) meeting the study inclusion criteria will be randomized for the study. See also Table of Study Procedures below.

Recruitment
Patients will be recruited at LRTC from institution's PTSD inpatient program by the study staff supervised by the On-site Investigator's team. Upon admission to the LRTC, patients will be informed about this treatment trial by either their staff psychiatrist or a member of the LRTC's research team. Each patient will be given an information sheet describing the study. If interested in participating, each patient will be shown a video describing the treatment trial and each of the tools/treatments used throughout the study (i.e. MRI and TMS).

Pre-Screening and LRTC Intake
Under an IRB approved HIPAA Waiver of Authorization, Waiver of Informed Consent, and Waiver of Documentation of Informed Consent, study personnel will conduct a brief pre-screening intake interview where the basic study inclusion and exclusion criteria will be reviewed to help the individual determine if he or she meets the study criteria or has obvious exclusions from the study protocol. This information will be maintained as a secure file with the following information: name, phone number, name of study, referral date, referral source, potential eligibility status, reason if not eligible, and verbal permission to contact the caller in the future for other studies. Subjects who agree to study participation will sign a consent document before any further screening will take place. Any individually identifiable information and Protected Health Information (PHI) collected on individuals who do not consent to participation will not become part of the research data. If subjects agree to participate in the research, the identifiable data collected will become part of the subjects' research records and will be stored according to the research confidentiality plan.

Consent Process
An authorized and trained member of the research team will engage the potential participant in an interactive explanation of the study guided by the informed consent document (ICD). After the participant has read the ICD, he or she will be given the opportunity to consider participation and discuss the research with family and friends. Once the potential participant has reached a decision, the advising staff member will review the purpose of study, duration of study, study procedures, the experimental components of the study, the potential risks and discomforts, the potential benefits, any alternatives to participation, protection of participant's confidentiality, and the contact information for both the researchers and the regulatory bodies overseeing the conduct of the study with the participant to ensure the participant has an understanding of the study. If the individual is agreeable to participation the advising staff member will then have the individual sign the consent form in the presence of a witness. A copy of the signed ICD will be given to the participant for their reference. Over the conduct of the study, the Research Team will be available to answer any questions about the research. Following consent, the participant meets regularly with the research staff. Ongoing discussions occur to ensure the participant's questions and concerns are addressed during the conduct of the study. Potential participants will have the study explained to them in a safe and private location before any assessments are conducted.

Phase I: Baseline Assessment. Following consent, a research team member will discuss the study with the participant again to fully explain the study procedures being conducted.

The participant will be asked to complete the following assessments:

1. *The Demographics and Military Service Characteristics Form* measures standard demographics (race, gender, age) and military service information (e.g., rank).

2. *History of Head Injuries (modified Defense and Veterans Brain Injury Center [DVBIC] 3-Item Screening Tool; Schwab, Baker, Ivins, Sluss-Tiller, Lux & Warden, 2006; Schwab, Ivins, Cramer, Johnson, Sluss-Tiller, Kiley, Lux & Warden, 2006).* We will use a modified version of the Defense and Veterans Brain Injury Center (DVBIC) 3-Item Screening Tool that was used in STRONG STAR. This instrument, initially called the Brief Traumatic Brain Injury Screen (BTBIS), was used as the gold standard for the diagnosis of TBI in a sample of soldiers returning from duty in Iraq and/or Afghanistan (Schwab, Ivins, et al., 2006). As recommended by the DVBIC, the 3-Question Screen will be considered positive when the participant endorses an injury (question 1) and altered consciousness (question 2, items A-E) for the worst head injury sustained while deployed. The form was modified for STRONG STAR and now CAP to capture the number of injuries, and to answer

question 2 based on the worst injury; the original form does not recognize the possibility of multiple head injuries during deployment. As the 3-Question Screen does not query head injuries prior to deployment, an additional four questions have been added to solicit information about each head injury sustained outside of deployment.

3. *Clinician-Administered PTSD Scale for DSM-5 (CAPS-5)* is a structured diagnostic interview and gold standard for assessing PTSD. It has excellent psychometric properties and diagnostic efficiency (Weathers, Keane, & Davidson, 2001). The scale also assesses social and occupational functioning, dissociation, and the validity of symptom reports. The CAPS was revised to accommodate the changes made in DSM-5, to reduce administration time, and to facilitate learning administration and scoring procedures. The CAPS for DSM-5 (CAPS-5; Weathers et al., 2013) now uses only a single 5-point ordinal rating scale to measure symptom severity. Symptom severity ratings combine information about symptom frequency and intensity obtained by the interviewer. CAPS-5 scores range from 0 to 80 with higher scores indicating greater PTSD symptom severity. At the same time, the CAPS-5 was revised with an eye toward maintaining backward compatibility with the DSM-IV version of the instrument. Because the measure is new, psychometrics and diagnostic cutoffs are still being evaluated. The CAPS-5 will be administered by certified independent evaluators (IE) blind to treatment status at the beginning and end of the intervention for all participants. Studies conducted near San Antonio and Fort Hood will be able to utilize IEs who receive training and ongoing support by the CAP Assessment Core. Remote studies will need to supply IEs for the various administrations of the CAPS-5. These staff will adhere to the Assessment Core's standardized procedures.

4. *PTSD Checklist for DSM-5 (PCL-5; Weathers, Litz, Keane, Palmieri, Marx, & Schnurr, 2013)* is similar in form to the PTSD Checklist (PCL) based on the DSM-IV (Weathers, Litz, Herman, Huska, & Keane, 1993). The PCL has excellent psychometric characteristics for screening and as a secondary indicator of PTSD symptom severity (see McDonald & Calhoun, 2010). The PCL-5 is a 20-item self-report measure, selected for its dimensional sensitivity, with higher scores reflecting greater PTSD severity. Scoring is based on how much the patient has been bothered by the symptoms in the past month on a scale from "0 = not at all" to "4 = extremely."

5. *Life Events Checklist for DSM-5 (LEC-5)* The LEC-5 (Weathers, Blake, Schnurr, Kaloupek, Marx, & Keane, 2013) includes the same list of 16 different potentially traumatic life events from the original LEC that are commonly associated with PTSD symptoms and designed to facilitate PTSD diagnosis (Gray, Litz, Hsu, & Lombardo, 2004). There is also a blank for specifying an additional stressful event not encompassed in the 16 events. For each potentially traumatic life event, respondents rate their experience of that event on a 6-point nominal scale (1 = happened to me, 2 = witnessed it, 3 = learned about it, 4 = part of my job, 5 = not sure, and 6 = doesn't apply). The primary addition to the LEC-5 is a category involving occupational exposure ("for example, paramedic, police, military, or other first responder"). There has not been a publication on the psychometric properties of the LEC-5, but the measure is nearly identical to the original LEC. In a group of 108 undergraduate psychology students the LEC demonstrated good convergence with the Traumatic Life Events Questionnaire (average kappa = 0.55) and correlated with the Posttraumatic Stress Disorder Checklist – Civilian version (reliability coefficients 0.34 to 0.48). The LEC demonstrated good test-retest reliability over 7 days. In 131 combat veterans the LEC was related in the predicted directions with other measures of psychopathology known to be associated with potentially traumatic life events as assessed by the Posttraumatic Stress Disorder Checklist – Military version, Clinician-Administered PTSD Scale, and the Mississippi Scale for Combat-Related PTSD.

6. *Deployment Risk and Resiliency Inventory-2 (DRRI-2) Combat & Postbattle Experiences subscale (Vogt, et al., 2013).* The DRRI-2 is a suite of 17 individual scales that assess key deployment-related risk and resilience factors with demonstrated implications for veterans' long-term health. The Combat Experiences and Postbattle Experiences subscales will be used to assess stressful deployment experiences.

7. *Patient Health Questionnaire-9 (PHQ-9; Kroenke, Spitzer, & Williams, 2001).* The PHQ-9 is a widely used and well-validated instrument for measuring the severity of depressive symptoms It consists of 9 items that assess both affective and somatic symptoms related to depression and depressive disorders; these 9 items correspond to the DSM diagnostic criteria for Major Depressive Disorder (MDD). Respondents rate the frequency with which they have been bothered by depressive symptoms within the past two weeks on a scale ranging from 0 ("not at all") to 3 ("nearly every day"). Scores on all items are summed to obtain a total severity score. Scores reflect no significant depressive symptoms (0-4), mild depressive symptoms (5-9), moderate depressive symptoms (10-14), moderately severe depressive symptoms (15-19), and severe depressive symptoms (>19). Respondents also indicate the degree to which their depressive symptoms have made it

difficult for them to do their work, take care of things at home, or get along with other people, from “not difficult at all” to “extremely difficult.” The PHQ-9 has high internal consistency (e.g., alpha ranging from .83 to .92; Cameron, Crawford, Lawton, & Reid, 2008), and correlates strongly with other measures of depression (Kroenke et al., 2001).

8. Structured Interview Guide for the MADRS (SIGMA)The MADRS is a standardized clinical assessment instrument to ascertain depressed mood and neurovegetative signs and symptoms of depression.

9. *Generalized Anxiety Disorder Screener (GAD-7; Spitzer, Kroenke, Williams, & Lowe, 2006).* The GAD-7 will be used to assess generalized anxiety symptomology. This is a 7-item measure that asks participants to rate the frequency with which they have been bothered by anxiety symptoms within the past two weeks on a scale ranging from 0 (“not at all”) to 3 (“nearly every day”). Scores on all items are summed to obtain a total severity score. Scores reflect no significant anxiety symptoms (0-4), mild anxiety symptoms (5-9), moderate anxiety symptoms (10-14), and severe anxiety symptoms (>15). Respondents also indicate the degree to which their anxious symptoms have made it difficult for them to do their work, take care of things at home, or get along with other people, from “not difficult at all” to “extremely difficult.” The GAD-7 has been shown to have high internal consistency (e.g., α = .89; Lowe et al., 2008) and has been shown to reliably discriminate between anxious and non-anxious diagnostic groups (Kroenke, Spitzer, Williams, & Lowe, 2010).

10. *Alcohol Use Disorders Identification Test (AUDIT) self-report version (Babor et al., 2001).* The AUDIT will be used to identify people with hazardous or harmful patterns of alcohol consumption and to index the severity of these problems. It will be administered as a self-report form. The AUDIT is a 10-item screening measure, developed by the World Health Organization (WHO), with three subscales (alcohol consumption, drinking behavior, and alcohol-related problems) that are scored on a 4-point scale for a highest possible total score of 40. The AUDIT has good internal consistency (αs = .80-.93) as well as sensitivity and specificity (Saunders, Aasland, Babor, De La Fuente & Grant, 1993; see Reinert & Allen, 2007, for review). The AUDIT’s time-frame is the last 12 months. Therefore, for trials without long-term follow-up, the AUDIT will be administered only at baseline. For studies with long-term follow-up (e.g., up to 12 months after the end of treatment), the AUDIT will be administered at both baseline and final follow-up.

11. *Quick Drinking Screen (QDS) self-report version (Sobell et al., 2003).* The QDS will be used to measure alcohol consumption. It consists of 4 items probing frequency and quantity of alcohol consumption. It will be administered in a self-report form. The QDS has been validated against the Timeline Followback daily estimation measure of alcohol use, and it shows good psychometric properties (Roy et al., 2008; Sobell et al., 2003). The QDS’s time-frame will be modified to match the “last two weeks” probed by the mandated depression and anxiety instruments for CAP studies (PHQ-9 and GAD-7). Like these other measures, the QDS can be administered frequently throughout CAP trials to track changes in alcohol use.

12. *Fagerstrom Test for Nicotine Dependence (FTND; Heatherton et al., 1991).* The Fagerstrom is a 6-item self-report measure that assesses severity of nicotine dependence. Questions probe both quantity of nicotine use (e.g., number of cigarettes per day) and pattern of use (e.g., time to first cigarette in morning). Respondents choose among response options, each of which is assigned a numerical value, with higher numbers corresponding to greater nicotine dependence. Scores on all items are summed to create a severity index with a range of 0 to 10, with higher scores indicating more severe dependence. The Fagerstrom scale has been shown to have high convergent validity with biochemical indices of nicotine use, and the measure has shown acceptable internal consistency (Heatherton et al., 1991). A review of 26 studies of the psychometric characteristics of the Fagerstrom found that it is a reliable instrument for measuring nicotine dependence in diverse settings and populations (Meneses-Gaya et al., 2009).

13. *Fagerstrom Test for Nicotine Dependence – Smokeless Tobacco (FTND-ST).* This is a modified version of the Fagerstrom Test that focuses on smokeless tobacco use, whereas the original Fagerstrom focuses exclusively on smoking. Like the FTND, the FTND-ST is a 6-item self-report measure of severity of nicotine dependence that has demonstrated convergent validity with biochemical indices of nicotine use (Ebbert et al., 2006; Ferketich et al., 2007). As on the original FTND, respondents choose among response options, each of which is assigned a numerical value, with higher numbers corresponding to greater nicotine dependence. Scores on all items are summed to create a severity index (range = 0–10).

14. *Insomnia Severity Index (ISI; Morin, 1993).* The ISI is a 7-item self-report measure that assesses perceived severity of insomnia. Each item uses a 5-point Likert type scale from 0 to 4, with higher numbers

corresponding to greater sleep problems. The items sum to produce a total score (range 0 – 28). The ISI has an internal consistency alpha coefficient of 0.74, and has shown convergent validity with other measures such as the Pittsburgh Sleep Quality Index (r = 0.67), the Dysfunctional Beliefs and Attitudes about Sleep (r = 0.55), and sleep diaries (r ranges from 0.32-0.91) (Bastien, Vallieres & Morin, 2001).

15. *Snoring, Tired, Observed, Blood Pressure (STOP) Sleep Apnea Screen (Chung et al., 2008).* To better understand sleep disturbance associated with PTSD and PTSD treatment, the STOP screen will be administered to screen for sleep apnea. The STOP is a four-item questionnaire developed and validated in 211 pre-operative surgical patients. Based on the endorsement of 2 or more questions, the sensitivity of the STOP ranged from 66% to 80% as compared with the apnea-hypopnea index (AHI) of polysomnography depending upon the AHI cut-off used. Individuals answering "yes" to 2 or more of the questions will be advised that they may be at risk for having sleep apnea and advised that they may want to speak with their primary care provider to consider referral for an overnight sleep evaluation.

16. *Patient Reported Outcomes Measurement Information System (PROMIS) Sleep Disturbance and Sleep-Related Impairment short forms (Yu, Buysse, & Germain, 2012).* The PROMIS Sleep Disturbance and Sleep-Related Impairment short forms are self-report measures of past-week sleep disturbance and past-week sleep-related impairment, respectively, derived from the larger PROMIS item banks (Buysse et al., 2010). Each short-form measure includes 8 items, with most items (symptoms) scored in intensity from 1 ("not at all") to 5 "(very much"). Each measure has shown strong reliability and construct validity (Yu et al., 2012).

17. *Depressive Symptom Index – Suicidality Subscale (Metalsky & Joiner, 1997).* The DSI-SS will be used to assess current suicidal ideation. The DSI-SS is a 4-item self-report measure of suicidal ideation that focuses on ideation, plans, perceived control over ideation, and impulses for suicide. It is being used as a core measure in the Military Suicide Research Consortium. Scores on each item range from 0 to 3, with higher scores reflecting greater severity of suicidal ideation. Instructions will instruct the participants to respond based on the past two weeks. A systematic review of measures of suicidal ideation and behaviors found that the DSI-SS had evidence of excellent internal consistency and concurrent validity (Batterham et al., 2014).

18. *Self-Injurious Thoughts and Behaviors Interview (SITBI; Nock, Holmberg, Photos, & Michel, 2007).* The SITBI is a structured interview assessing the historical presence, frequency, and characteristics of self-injurious and suicidal thoughts and behaviors. The short form version of the SITBI, with 72 items total if no skip-outs are used (i.e., the patient endorses the initial item in each module), will be administered at baseline by an Independent Evaluator, who will instruct the participants to answer the questions based on their entire lifetime of experience. The SITBI has shown high interrater reliability, test-retest reliability, and concurrent validity (Nock et al., 2007).

19. *Veterans RAND 12-Item Short Form Health Survey (VR-12).* The VR-12 is a 12-item health questionnaire that was developed from, and explains 90% of the reliable variance of, the longer VR-36 (Kazis, Selim, Rogers, Ren, Lee, & Miller). Its items are sampled from each of the eight health domains from the VR-36: physical functioning, role limitations due to physical problems, bodily pain, general health perceptions, energy/vitality, social functioning, role limitations due to emotional problems, and mental health. Also, there are two summary scales: a physical component summary (PCS) and a mental component summary (MCS). Each item includes a 5-point response scale ranging from "no, none of the time" to "yes, all of the time." The VR-36 has been widely used, distributed and documented in the Veterans Health Administration. Higher scores indicate better health.

20. *Brief Inventory of Psychosocial Functioning (B-IPF).* This is a 7-item self-report instrument measuring respondents' level of functioning in seven life domains: romantic relationship, relationship with children, family relationships, friendships and socializing, work, training and education, and activities of daily living (Marx, 2013). Respondents indicate the degree to which they had trouble in the last 30 days in each area on a 7-point scale ranging from "0 = Not at all" to "6 = Very much." The B-IPF has demonstrated concurrent validity, and the full 80-item IPF from which it was created has strong test-retest reliability and internal consistency (Marx, 2013).

21. *Health Questionnaire.* The Health Questionnaire measures medical and mental health diagnoses that respondents have received, medical board and disability status, medications taken, and caffeine use. The version of the Health Questionnaires used at follow-ups also probes emergency room use, hospitalizations, mental health treatments, military status changes, and any important new life events or changes since the time of the last assessment.

22. *Clinical Global Impressions (CGI; Guy, 1976)* The Clinical Global Impression rating scales are commonly used measures of symptom severity, treatment response and the efficacy of treatments in treatment studies of patients with mental disorders. Many researchers, while recognizing the validity of the scale, consider it to be subjective as it requires the user of the scale to compare the subjects to typical patients in the clinician experience. The Clinical Global Impression – Severity scale (CGI-S) is a 7-point scale that requires the clinician to rate the severity of the patient's illness at the time of assessment, relative to the clinician's past experience with patients who have the same diagnosis. Considering total clinical experience, a patient is assessed on severity of mental illness at the time of rating 1, normal, not at all ill; 2, borderline mentally ill; 3, mildly ill; 4, moderately ill; 5, markedly ill; 6, severely ill; or 7, extremely ill. The Clinical Global Impression – Improvement scale (CGI-I) is a 7 point scale that requires the clinician to assess how much the patient's illness has improved or worsened relative to a baseline state at the beginning of the intervention. It is rated as: 1, very much improved; 2, much improved; 3, minimally improved; 4, no change; 5, minimally worse; 6, much worse; or 7, very much worse. The Clinical Global Impression – Efficacy Index is a 4 point × 4 point rating scale that assesses the therapeutic effect of the treatment as 1, unchanged to worse; 2, minimal; 3, moderate; 4, marked by side effects rated as none, do not significantly interfere with patient's functioning, significantly interferes with patient's functioning and outweighs therapeutic effect.

Phase I: Baseline MRI. Once the participant has completed the psychological assessments required to determine inclusion/exclusion criteria, eligible participants will be taken from the LRTC to the Research Imaging Institute (RII) at UTHSCSA. When the participant arrives, the participant will undergo an anatomical and functional magnetic resonance imaging (MRI) session. Earplugs will be provided at every MRI session. MRIs use strong magnets and radio waves to non-invasively acquire images of organs and tissues. Unlike X-rays, MRIs do not expose subjects to radiation, therefore it is considered safe to obtain many MRIs in a single scanning session or over multiple days; the initial MRI scans may take up to 2 hours. In the event that the participant cannot tolerate the MRI procedure, they will be withdrawn from the study. Participants will be imaged at the RII using a research-dedicated 3T Siemens TIM/Trio MRI scanner (Siemens, Erlangen, Germany) with an eight-channel phased array coil. Structural imaging will be obtained only once, upon enrollment. Structural images will be used for irTMS treatment planning (described below) and for spatial normalization to a standard anatomical template. MRI images will be obtained two times (i.e. pre-treatment and post-treatment) and used to detect symptom-specific network abnormalities and treatment-induced plasticity. We will also perform motor threshold assessments for each patient (at pre-treatment and post-treatment, immediately after each MRI) to determine the correct intensity and train durations needed to safely deliver irTMS in each patient's 3-Week Treatment Phase.The post-treatment motor threshold assessment are important to the future establishment of safety guidelines for irTMS treatment of PTSD.

Structural MRI. T1- and T2-weighted structural MRIs will be used for treatment-plan computation (described below) and for spatial normalization of functional images. T1-weighted images are obtained in 3D modes (1.0 mm cubic voxels) with acquisition parameters of: repetition time (TR) = 1900 ms, echo time (TE) = 2.26 ms, tip angle = 9˚, slice thickness = 1.0 mm, in-plane resolution = 1.0 x 1.0 mm^2 acquiring 256 volumes. T2-weighted images will be obtained with and without fat suppression (turbo spin echo, 34 axial slices, matrix size = 640 x 640, voxel size = 0.4 x 0.4 x 5.2 mm^3, flip angle = 120°, TR/TE = 14000/93 ms, turbo factor 18). Total structural MRI scan time is approximately 20 minutes. To ensure accurate irTMS treatment planning, we will also acquire diffusion tensor images (DTI). DTI will be obtained from the diffusion-weighted images using a single-shot echo-planar gradient-recalled echo, T2-weighted, sequence to acquire diffusion-weighted data with the spatial resolution of 2.0 x 2.0 x 2.0 mm and TE/TR = 85/9300. The diffusion-weighted scans will have 64 different non-collinear diffusion sensitization directions, consisting of diffusion-weighted images at b = 700 mm^2/s and one non-diffusion weighted image at b = 0 mm^2/s. The total DTI sequence acquisition time will be 20 min.

BOLD fMRI for motor threshold assessment. Functional images will also be used to identify each subject's primary motor cortex for motor threshold assessment. BOLD fMRI will be acquired in a block design paradigm utilizing gradient echo planar images with the following parameters: TR = 2.5 s, TE = 30 ms, a flip angle of 90º, and slice thickness = 4.0 mm. Thirty-six continuous slices will be acquired, with an in-plane spatial resolution of 1.7 x 1.7 mm^2. During the BOLD fMRI scan, participants will perform abduction and adduction of the right index finger (in a block design); this has been shown to maximally activate the first dorsal interosseous muscle area of the hand's motor cortex. The task and rest periods are 50 seconds long and are interleaved during the 6 minutes of data acquisition. During the rest period, the participants will be instructed to remain motionless, to keep their eyes open, and to perform no behavioral task.

Intrinsic Connectivity by Blood-Oxygen-Level-Dependent (BOLD) fMRI for computing treatment plans. Intrinsic connectivity networks will be measured using blood oxygen dependent (BOLD) fMRI. BOLD fMRI is the most

robust and widely used pulse sequence for intrinsic connectivity modeling. BOLD images will be used for computing treatment plans and for testing for intrinsic connectivity changes by symptom score and with treatment. BOLD data will be acquired as 360 whole-brain volumes acquired with a TR = 2500 ms, TE = 30 ms, tip angle = 90˚, 128 x 128 voxels, 1.7 mm x 1.7 mm, and 36 slices (4.0 mm thickness). This sequence requires ~ 15 minutes. Resting-state BOLD (15-min acquisition) will be used to map per-subject the voxel-wise intrinsic connectivity of DLPFC with the anterior cingulate cortex (ACC) and Precuneus; this will guide aiming parameters.

Motor Threshold Assessment. After the baseline MRI session, each patient will undergo a brief motor threshold assessment by single pulse TMS to their primary motor cortex hand region which elicits a contraction of the targeted hand muscle. The motor threshold assessment begins by locating the hand region and applying single pulses of TMS—with at least 5 seconds between successive pulses—at increasing levels of TMS intensity. We continue to ramp up the TMS intensity with each TMS pulse until we observe a contraction of the targeted hand muscle in 5 out of 10 pulses at a given TMS intensity. This TMS intensity will then be recorded as that patient's resting motor threshold. Using the patient's BOLD fMRI of the primary motor cortex, we can calculate their coil-to-cortex depth and use this information to determine the correct irTMS treatment intensity for the DLPFC and also determine the correct train duration needed to safely deliver 20Hz irTMS in each patient using published safety guidelines on appropriate train durations at specific TMS frequencies and intensities.

Random Assignment. Prior to the Treatment Phase, participants will be randomly assigned using a statistically generated randomization program to 1 of 2 active irTMS treatment arms. An irTMS treatment plan will be developed specifically for each participant, which will be securely-transmitted to the irTMS treatment team at the LRTC. The master list of treatment arm enrollment will be securely-stored (i.e. password-encrypted) on UTHSCSA's XNAT server and will not be available to members of the research team until all of the participant's treatment sessions and symptom assessments have been completed. Therefore, all members of the LRTC's irTMS treatment team will be blinded to which treatment arm each participant is assigned. irTMS treatment visits will take place at the LRTC immediately following the Baseline scan and development of the treatment plan.

Phase II: 3-Week Treatment Phase.

irTMS Treatment Planning. All irTMS treatments will be planned for robotic, image-guided irTMS delivery—which will be performed at the LRTC. The acquired MRI images will be used to compute a patient-specific, treatment plan, which will be stored and used during their respective irTMS session at the LRTC. Image guidance will be based on treatment plans developed from high-resolution structural MRI and resting-state BOLD fMRI acquired at the RII. From the T1-weighted MRI (described above), two surface models are computed: a model of the scalp surface, for use in registration of the patient to the treatment plan; and, a model of the cortical surface including cortical surface normals. Both models are created using a modification of the dividing cubes algorithm following automated tissue segmentation. The scalp surface model is based on the air-scalp interface, which is readily obtained by simple thresholding. The cortical surface model is based on pre-classification of brain tissue into gray, white and CSF surfaces, using the FMRIB's Automated Segmentation Tool.

The right DLPFC treatment target locations will be based on connectivity strength with ACC and Precuneus using region-seeded resting-state BOLD fMRI. Coil orientation will be corrected for the underlying gyral anatomy, using a T1-MRI derived cortical surface model to align the E-field according to the cortical column cosine model. Stimulation intensity will be corrected for the distance of the stimulation site from the scalp/coil surfaces. Intensity ranges will be individually adjusted for each patient—to deliver depth-corrected E-fields between 80 volts/meter and 95 volts/meter.

<u>Sham TMS</u>: An image-guided treatment plan will be computed for sham TMS subjects in the same manner as for active irTMS to ensure therapist blinding.

irTMS Treatment Sessions. All participants will continue with their TAU at LRTC (or its treatment clinics) including regular medication management encounters with their attending psychiatrist and therapy sessions as of their treatment. An effort will be made to limit the disruptions in patients' TAU by working research assessments and irTMS around their expected TAU programing. irTMS treatment visits will take place at the LRTC starting as soon as possible after the treatment plan is completed, expected to be 1-2 working days following baseline scanning.

irTMS Position/Holding. For each subject, the coil pose will be pre-computed from structural and functional MRI and stored as an individualized treatment plan. Each subject's treatment plan will be registered to his/her head by creating a scalp model with a high-resolution digitizer, an integrated component of the irTMS system. In each arm,

the TMS coil will be positioned and held by the robot (under the treatment technician's supervision).

irTMS Treatment Protocol. At the respective TMS treatment target site, the LRTC team will deliver irTMS using an irTMS treatment protocol of PTSD which falls within the previously published safety guidelines for TMS treatments. This TMS protocol consists of 1,600 irTMS pulses per session, delivered at a frequency of 20 Hz. Intensity ranges (designed to deliver depth-corrected E-fields of 80-95 volts/meter) will be determined when the patient's treatment plan is developed and these intensity ranges will be given to the treatment technicians with the irTMS treatment plan. On the first day of the irTMS treatment, the patients will be given brief trains of TMS pulses beginning at 95 volts/meter of intensity. If that level of intensity causes discomfort or significant levels of facial muscle twitching, the intensity will be adjusted downward in 5 volt/meter units (i.e., 90, 85, 80 etc.) until a level is found within the 80-95 volts/meter range. That final intensity will be used throughout the remaining treatment phase for that patient. If study participants decide that they cannot tolerate the lowest prescribed dose of irTMS (e.g. 80 volts/meter), they will be withdrawn from the study (see Discontinuation and Withdrawal of Patients Section below).

TMS treatment guidelines recommend that the train duration of TMS pulse protocols remain within the limits of published safety guidelines (reached by a consensus of TMS practitioners). For example, if a patient requires a TMS intensity equivalent to 130% of their motor threshold, then we must limit our TMS train durations to 0.55 seconds (i.e. 11 pulses/train). Therefore, we will adjust the train duration of each patient's treatment protocol so that it falls within the limits of these recommendations. We will also adjust the intertrain interval to ensure that we deliver all of our treatment pulses within the hour-long treatment session. For example, if a patient needs a TMS intensity equivalent to 130% of their resting motor threshold, we will deliver 160 pulse trains of irTMS (e.g. train duration = 0.5 seconds; intertrain interval = 7.5 seconds; 10 pulses per train), which would require 21.33 minutes of treatment time to deliver the desired number of TMS pulses in each session (i.e. 1,600 pulses). The primary TMS treatment parameters (TMS frequency (i.e. 20Hz), number of pulses per session (i.e. 1,600), and number of sessions (i.e. 20) will remain the same across patients. We may only adjust the patient's intensity and the protocol's train duration and intertrain intervals so that they fall within the safety limits of repetitive TMS treatments.

Active treatment will use a liquid cooled figure-8 MagPro coil (MagVenture, Denmark). Sham treatment will use a coil that simulates the sound and scalp stimulation of TMS but does not deliver an E field (MagVenture, Denmark).

Participants will receive treatment 7 days per week for as long as they are hospitalized at LRTC for up to 20 sessions. On each treatment day, a total of 1,600 pulses will be administered within the one hour TMS session. With the set up for treatment, the total time required for each treatment visit should be less than one hour per day. This protocol will administer a total of 24,000 pulses over the three-week period. If study participants decide, at any time, that they cannot tolerate the irTMS treatments, they will be withdrawn from the study (see Discontinuation and Withdrawal of Patients Section below) and will be assisted in finding appropriate after-study care.

Phase II: Post-Treatment Follow-Up

Post-treatment MRI. Immediately following the last irTMS treatment at the end of the 3rd week, a second functional MRI scan will be conducted to assess treatment effects. The post-MRI will occur any time after last treatment with up to a 3-day window for completion. In rare instances, if scheduling circumstances (such as weekends and institution holidays, MRI staff outages, participant discharge date) prevent the post-MRI from occurring after all treatment sessions have been completed, the post-MRI may be conducted before the final treatment session, and any remaining treatment sessions will occur after the post-MRI. This will be documented in the participant study file and will be considered during data analysis. After the post-treatment MRI session, each patient will also undergo another brief motor threshold assessment in order to document any changes which may be used to report on the safety of this TMS treatment protocol. This second scanning visit should take approximately 1 hour.

Participant Blind Form. The Participant Blind Form measures the participant's best guess about the study treatment that they received, level of confidence in this guess, and how unblinding could potentially have occurred. This form will be used at treatment completion and follow-up for all CAP RCTs that are double-blind (i.e., medication studies).

Independent Evaluator Blind Form. The Independent Evaluator Blind Form measures the independent evaluator's best guess about the study treatment that the subject received, level of confidence in this guess, and how unblinding could potentially have occurred.

Phase III: 1 Month/3 Month Follow-Up
One month and three months after the final irTMS treatment session, participants will be interviewed (either in-person or over the telephone) by a member of the research team. Self-report measures may be administered over the phone or by direct electronic data capture via a secure link to the secure STRONG STAR database.

Table of Study Procedures:

Phase	Pre-Study (at LRTC)			3 Week Treatment (at LRTC & the RII)			Post-Treatment (at the RII)	1 Month/ 3 Month Follow-Up (either in-person or via telephone)
Week	Wk 0			Wk 1	Wk 2	Wk 3	Wk 3	Wk 7/Wk15
	LRTC Intake	Baseline	MRI Appt					
Day(s)	0 to 1	1-3	2-3	7	14	20	21 -24	50/110
Medical History	X							
Metal Screening		X						
Informed Consent		X						
1. Demographics & Military Service Characteristics		X						
2. History of Head Injuries Screening		X						
3. Clinician-Administered PTSD Scale for DSM-5 (CAPS-5)		X						X
4. PTSD Checklist for DSM-5 (PCL-5)		X		X	X	X		X
5. Life Events Checklist - 5 (LEC-5)		X						X
6. Deployment Risk and Resiliency Inventory-2 (DRRI-2) Combat and Postbattle Experiences Subscales		X						
7. Patient Health Questionnaire-9 (PHQ-9)		X		X	X	X		X
8. 'Structured Interview Guide for the Montgomery-Ã…sberg Depression Rating Scale (SIGMA).pdf'		X				X		X
9. Generalized Anxiety Disorder Screen (GAD-7)		X		X	X	X		X
10. Alcohol Use Disorders Identification Test (AUDIT) self-report version		X						X
11. Quick Drinking Screen (QDS) self-report version		X						X
12. Fagerstrom Test for Nicotine Dependence		X				X		X

13. Fagerstrom Test for Nicotine Dependence – Smokeless Tobacco		X				X		X
14. Insomnia Severity Index (ISI)		X				X		X
15. Snoring, Tired, Observed, Blood Pressure (STOP) Sleep Apnea Screen		X						
16. Patient Reported Outcomes Measurement Information System (PROMIS) Sleep Disturbance and Sleep-Related Impairment short forms		X				X		X
17. Depressive Symptoms Index – Suicidality Subscale		X				X		X
18. Self-Injurious Thoughts and Behaviors Interview short form		X				X		X
19. Veterans RAND 12-Item Short Form Health Survey (VR-12)		X						X
20. Brief Inventory of Psychosocial Functioning (B-IPF)		X						X
21. Health Questionnaire		X				X		X
22. Clinical Global Impressions (CGI)		X				X		X
Adverse Events Monitoring			X	X-X	X-X	X-X		X
Concomitant Medications/Treatment	X-X	X-X		X-X	X-X	X-X		X
Structural MRI			X					
Intrinsic Connectivity by Blood-Oxygen-Level-Dependent Functional MRI			X				X	
Motor threshold assessment			X				X	
irTMS Treatment (20 consecutive days)				X-X	X-X	X-X		
Participant Blind Form							X	
Independent Evaluator Blind Form								X

<u>Data Protection</u>. Data will be coded using an assigned number. Data collected during treatment will be placed into locked cabinets at the LRTC offices. Audio tapes will be uploaded to the secure STRONG STAR data base. Every member of the research team will be trained and monitored about how to handle and protect both medical and research records. Furthermore, the research team strictly controls access to study data. No identified data (either copies or originals) will be maintained at the study site. Local study site will maintain a list of assignment numbers for the purpose of linking subsequent research materials. All UTHSCSA STRONG STAR network connectivity is segmented with Access Control Lists and is not accessible to any other UTHSCSA network segments. STRONG STAR data server is physically located at the Advanced Data Center (ADC) has 24x7 onsite security, card key access controls and video surveillance. University of Texas Health Science Center at San Antonio (UTHSCSA) ADC facility also maintains Gen 2 firewall devices to protect and prohibit any unauthorized access to UTHSCSA data. All UTHSCSA network devices are monitored by state of the art monitoring applications that include configuration audit, management, and availability 24x7.

The UTHSCSA STRONG STAR data server is currently a VMware Instance running Windows

Server 2016 Enterprise Standard with daily backup services and vSphere Business Continuity Advanced Failover.

Only select Data Core personnel have direct access to the data on a "need to access basis". Data Core also follows the Principals Of Least Privilege (POLP). For example (but not limited to) detecting and repairing data corruption and producing reports not currently within the STRONG STAR system. All user activity is tracked and recorded within the system so if any records are added, altered or viewed the action is recorded and can be recalled for auditing purposes. Access to this information will require a password-protected login available only to authorized Core staff.

<u>Risk/Benefit Assessment.</u>

Risks and Side Effects related to the irTMS applied to Dorsolateral Prefrontal Cortex Brain Region:

- Likely, some may be serious. In 100 people, approximately 20-30 may have:
  - Mild headache
  - Scalp and/or Facial pain/discomfort
  - Scalp and/or Facial Muscle Twitching
  - Neck pain
- Less Likely, some may be serious. In 100 people, approximately 5-10 may have:
  - Dizziness
  - Sleepiness
- Rare and Serious. In 100 people, approximately 1-2 may have:
  - Seizures

Risks and Side Effects related to the Robot placement of the irTMS include those which are:

- Rare and Serious. In 100 people, approximately 0-1 may have:
  - Physical injury from the robotic device.

Risks and Side Effects related to the MRI include those which are:

- Rare and Not Serious. In 100 people, approximately 1 may have:
  - Claustrophobia (fear of tight spaces). During the MRI scan, participants will be asked to remain perfectly still.
- Rare and Serious. In 100 people, approximately 1-2 may have:
  - Foreign bodies which may interact with the magnetic field of the MRI

Risks related to the Psychological Assessments include those which are:

- Less likely (less than 5-20 subjects out of 100) and Not Serious:
  - Emotional distress including experiencing an initial increase of PTSD symptoms due to the discussion of traumatic events.
- Rare (less than 5 subject out of 100) and Serious:
  - Breach of confidentiality.

Risks of having a diagnosis of PTSD regardless of participation in this research or not: Individuals with PTSD may have suicidal thoughts or attempt suicide. This is a risk to you whether you are being treated for PTSD or not. Therefore, the risk of suicide is not any higher in the study than it would be if you were not in this study.

<u>Risk Minimization</u>.

- To physical injury by the robotic device. Robotic devices must be used with caution in the presence of persons. The robotic device used here (KUKA®) poses no significant risk. The KUKA® moves slowly, so that it can easily be stopped prior to any collision. Loss of electrical power renders the KUKA® robot immobile in its current position, (i.e., it does not return to a "parking" position or make any sudden motions). The KUKA® robot requires a key and a recessed button to actuate, being used only under direct supervision. Collectively, these features make the KUKA® robot safe for use by and with humans.
- To claustrophobia in the MRI.
- To injury during the MRI from foreign bodies interacting with the magnetic field. MRI can have risk for persons with foreign bodies implanted in their body. Cardiac pacemakers and cochlear implants may cease to function and can be permanently damaged by the MRI. Surgical clips on aneurysms and intestines may be moved by the magnetic field. Ferrous metal filings in the eye (e.g., in machinists) can be

	moved by the magnetic field. Foreign body risk is minimized by including only volunteers with no known foreign bodies and no exposure to circumstances, which might predispose to foreign bodies (e.g., metal machine workers). Before receiving an MRI, participants will be asked about any metal objects that may be in their body.<br>• To emotional distress during psychological assessments. All clinical staff associated with assessment have experience in psychiatric evaluation and will implement protocol procedures in a sensitive and supportive manner. Interviews will be stopped if subjects become distressed or object to answering questions. Measures implemented to minimize risks are: 1) Before participating, subjects undergo careful psychiatric and medical evaluation. 2) An experienced study clinicians are available at all times during the sessions to provide medical a psychiatric monitoring for subjects experiencing any difficulties during treatment. 3) All subjects will be seen weekly for ongoing assessment of behavioral symptoms. 4) If a subject's clinical condition significantly worsens, he or she will be withdrawn from the research protocol to continue TAU at LRTC. Any indication that a participant's suicidality has worsened from baseline will be handled using processes at the LRTC.<br>• To breach of confidentiality. Study participants will be treated in individual offices located on the LRTC. Data a will be stored by an assigned participant code number so that data records and specimens can be viewed by password-authenticated, authorized investigators and Consortium personnel. Digital audio recordings of assessments will be labeled with the participant's study id number and saved on a secure password protected server. Those recordings to be reviewed for fidelity to ensure that the assessment is being delivered in accordance with Consortium standardized procedures. There is no option for the reviewers to download or otherwise save the recordings to their computers. Every member of the Research Team will be trained and monitored about how to handle and protect both medical and research records. Only authorized study staff, and members of the Data Management and Biostatistics Core will have access to either the raw data or electronic study data.<br><br>Discontinuation of Subjects. Subjects may be discontinued from study treatment and assessments at any time. Specific reasons for discontinuing a subject from this study are: 1) Voluntary discontinuation by the subject, who is at any time free to discontinue his/her participation in the study without prejudice to further treatment; 2) Clinical deterioration: The following are objective criteria for clinical deterioration, (a) a 25% increase in the PCL-5 at any time during the study, (b) Patients with a CGI-I score ≥ 6 at any post-baseline visit, and (c) the onset of active suicidality as assessed by the study clinicians requiring treatment outside of the study protocol; 3) Evidence of intolerable adverse reaction, or unable to tolerate irTMS or MRI; 4) Safety reasons as judged by the investigator.<br><br>**Potential Benefits.**<br>• The potential benefit to participants is the potential for PTSD and/or depressive symptom relief.<br><br>**Adverse Events, Unanticipated Problems, and Deviations.**<br>Adverse Events will be assessed and monitored according to the established STRONG STAR and CAP SOPs and the IRB of record's policies and procedures. Reporting Adverse Events, Unanticipated Problems Involving Risks to Subjects or Others (UPIRSOs), and Deviations to the Office of the IRB. All adverse events, unanticipated problems involving risk to subjects or others, and deviations will be reported to the Institutional Review Board (IRB) in accordance with current IRB policy. UPIRSOs and recurrent non-compliance with study procedures will be reported promptly to the IRB. All adverse events that do not meet the UPIRSO criteria and deviations that are not non-compliance will be summarized at Continuing Review per the IRB of record's policy.

Research Monitor.

The study Research Monitor, Karen Nijland, BPharm, will oversee the safety of the research and report observations/findings to the IRB or a designated institutional official. The IRB assigned Research Monitor's duties include: Act as an advocate regarding complaints or concerns from subjects; verify the IRB approved safety monitoring plan is being followed; verify that UPIRSO determinations are made in a timely fashion; and concurrently review UPIRSO reports submitted by PI to the IRB. In addition, the Research Monitor will review all unanticipated problems involving risks to subjects or others associated with the protocol and provide an independent report of the event to the IRB. The Research Monitor may discuss the research protocol with the investigators, interview human subjects, and consult with others outside of the study about the research; shall have authority to stop a research protocol in progress, remove individual human subjects from a research protocol, and take whatever steps are necessary to protect the safety and well-being of human subjects until the IRB can assess the monitor's report; and shall have the responsibility to promptly report their observations and findings to the IRB or other designated official and the HRPO.

Data Analysis

Neuroimaging Data Analysis Plan: *Structural MRI: The* T1- and T2-weighted images will be pre-processed using FSL's Brain Extraction Tool (Smith, 2002), then segmented into three tissue classes: gray, white and ventricular components, using the FMRIB's Automated Segmentation Tool (FAST; Zhang et al., 2001). Acquisition of both the T1- and T2-weighted images allow more accurate tissue segmentation and TMS treatment planning; the gray-matter surface normal will be used for treatment planning. Images will be spatially normalized to Montreal Neurological Institute space for group-wise analyses. The DTI data will be pre-processed using FSL's diffusion toolbox.

BOLD fMRI for motor threshold assessment. Each subject's BOLD fMRI will be analyzed using FSL's FEAT program to determine his/her primary motor cortex targets for motor threshold assessment. This information will be used to determine the TMS intensity needed for each patient's irTMS treatment plan and it will also inform the train durations of each treatment session so that the intensity of these pulses falls within the published safety limits of TMS delivered at 20 Hz.

Intrinsic Connectivity by BOLD fMRI. Data will be analyzed using FSL's FEAT program. Head movements are corrected by affine registration using a 2-pass procedure. BOLD data will be normalized to MNI space single-patient template using the "unified segmentation" approach, followed by a 5-mm full width at half maximum Gaussian smoothing. Spurious correlations with nuisance variables are removed using a recently validated framework. Data are band-pass filtered preserving frequencies between 0.01 and 0.08 Hz. The time course of each seed region's BOLD signal will be extracted as the first eigenvariate of activity in all gray-matter voxels located within the respective cluster. To quantify resting-state functional connectivity linear (Pearson) correlation coefficients between the time series of the seeds (targeted DLPFC nodes of each patient's DLPFC-limbic and DLPFC-motor networks) and any other gray matter voxel will be computed. Voxel-wise correlation coefficients will be transformed into Fisher´s Z-scores (with non-sphericity correction). These images will be used for irTMS treatment planning. The resting-state fMRI scan will also be used to assess, network-specific functional connectivity differences between each patient's pre-treatment and post-treatment scans.

Clinical Outcomes Data Analysis plan. All pre-treatment and post-treatment MRIs will be acquired and analyzed at the RII, however, all of the statistical analysis of the symptom scores for this project will be done upon completion of recruitment and termination group blinding. The primary efficacy outcome measure will be the PTSD Checklist (PCL-5) for symptoms assessed pre-treatment, weekly during treatment, post-treatment, and 1-month following the completion of treatment and the Clinician-Administered PTSD Scale for DSM-5 (CAPS-5) for the diagnosis of PTSD assessed pre-treatment and 1-month following the completion of treatment. The primary analysis will use mixed effects regression models. Secondary analysis will focus on changes in PTSD symptoms clusters, effects of the intervention in mood symptoms, functioning and cognitive outcomes. Types and rates of adverse events will be summarized and compared across the study groups. The durability of benefit and the clinical significance of PCL changes will be explored. Non-compliance to protocol as defined as missing two consecutive irTMS sessions or receiving less than 16 irTMS sessions during hospitalization at LRTC will be assessed for and evaluated in data analysis.

STATISTICAL ANALYSIS PLAN

Residential Therapy with Navigated TMS for Combat-Related Posttraumatic Stress Disorder: A Randomized Clinical Trial.

Overview

This Statistical Analysis Plan (SAP) describes the analytic approach for clinical outcomes in a randomized, sham-controlled trial in which participants received standard-of-care therapy (SoC) plus either active transcranial magnetic stimulation (SoC + Active) or sham stimulation (SoC + Sham). Analyses were conducted using linear mixed-effects models (LMMs) that compared the two randomized groups (SoC + Active and SoC + Sham) across time (Chakraborty & Gu, 2009; Little et al. 2012). .

Analytic sample

Analyses used the full intention-to-treat (ITT) sample (N=119). Of the enrolled participants, six (5.04%) were complete dropouts with no follow-up data, and 91 (76.47%) had partially missing data at one or more assessment points. Of 714 possible total observations for the PCL-5 (i.e., 119*6), 186 (26.04%) were missing. LLMs were estimated using all available observations for each participant. Participants with incomplete follow-up contributed available data without listwise deletion. No imputation was performed. The restricted maximum likelihood (REML) estimation was used with these LLMs, allowing for unbalanced repeated-measures data and the inclusion of participants with incomplete measurements under the missing-at-random (MAR) assumption. The MAR assumption posits that missingness may be related to observed variables (e.g., higher baseline PTSD scores predicting dropout) but not to unobserved values of the outcome itself after accounting for observed covariates. This approach is appropriate for handling dropout patterns associated with measured baseline characteristics and leverages modern missing-data methods that yield unbiased estimates under MAR.

Outcomes and assessment schedule

Primary outcome. Change in PTSD symptom severity measured by the PTSD Checklist for DSM-5 (PCL-5) from baseline to end of treatment (EoT; three weeks post-baseline for the PCL-5).

Secondary outcomes. Change in Clinician-Administered PTSD Scale for DSM-5 (CAPS-5) and depressive symptoms measured by the Patient Health Questionnaire-9 (PHQ-9), evaluated across the Treatment Phase and Follow-up Phase.

PCL-5 and PHQ-9 measurements were obtained at six timepoints: baseline; weekly during the 3-week treatment period, with the week 3 measurement serving as EoT; and 1-month and 3-month follow-ups (1MFU and 3MFU). CAPS-5 scores were obtained at baseline, 1MFU, and 3MFU, with the 1MFU measurement serving as the EoT assessment for the CAPS-5.

Effect size calculations

Standardized effect sizes were reported as Hedges' *g* for (a) between-arm differences at endpoints of interest and (b) within-arm change from baseline to endpoints of interest. Hedges' *g* applied the small-sample correction to the standardized mean difference. Pooled standard deviations based on observed values were calculated as appropriate for the Hedges' *g* denominator (i.e., either across arms at the endpoint of interest, or across baseline and endpoint measurements for the arm of interest). The pooling of standard deviations accounted for sample size differences across time points.

General modeling framework

Clinical outcomes were analyzed using LMMs (two-level multilevel linear models) with repeated observations nested within participants. Outcomes were treated as continuous and modeled with an identity link. The treatment indicator variable was coded 0 = SoC + Sham and 1 = SoC + Active. Models included random intercepts for participants and fixed effects for Arm, Time, and the Arm x Time interaction. No additional covariates were included. Measurement residuals were assumed normally distributed with constant variance.

A piecewise approach to modeling was used for hypothesis testing for each outcome. Models for EoT outcomes included all available measurements from baseline to EoT. For the PCL-5 and PHQ-9, these were the baseline, week 1, week 2, and week 3 (EoT) measurements. For the CAPS-5, these were the baseline and

1MFU (EoT) measurements. Models for 3MFU outcomes for the PCL-5 and PHQ-9 included the EoT, 1MFU and 3MFU measurements. For the CAPS-5, the model for 3MFU scores included the 1MFU (EoT) measurement.

Based on inspection of observed means, the effect of time was initially modeled as linear in each piecewise model. The assumption of linearity was checked by comparing fits with models that treated time as nonlinear (e.g., quadratic or cubic functions), given the number of available time points. For each model, the linear function demonstrated the best fit.

Time coding and intercepts

Time was coded ordinally by measurement occasion and centered so that the intercept corresponded to the endpoint of interest in each analysis. For example, in the primary analysis comparing PCL-5 scores of the two Arms at EoT, baseline, week 1, week 2, and week 3 (EoT) measurements were coded as -3, -2, -1, and 0, respectively. For the secondary analysis comparing PCL-5 scores of the two Arms at 3MFU, the 3MFU, 1MFU, and EoT measurements occasions were coded as 0, -1, and -2, respectively. Both models for CAPS-5 outcomes thus included two time points each; the EoT model included baseline (-1) and 1MFU (0) measurements, and the 3MFU model included 3MFU (0) and 1MFU (-1) measurements. Centering in this way allows the main Arm effect in the LMMs to directly estimate the between-arm difference at the endpoint of interest.

Primary hypothesis and decision rule

For both primary and secondary outcomes, two estimates were of key importance. The first is the modeled difference in treatment arm means at the occasion of interest (EoT or 3MFU), corresponding to the coefficient/main effect of Arm as parameterized in the model. Also of relevance is the modeled difference in slope between the two arms, corresponding to the Arm*Time interaction coefficient. The former provides an effect estimate and associated inferential test of whether the two treatment conditions resulted in different outcome means at the occasion of interest. The latter provides an effect estimate and inferential test of whether the two arms differed in trajectory over the period of interest.

One-sided p-values were used. Unless otherwise specified, statistical significance was evaluated at alpha = 0.05 (one-sided). No multiplicity adjustment was applied; secondary outcomes and follow-up phase analyses were interpreted as supportive/exploratory.

Reliable Change Index. As a supplement to the primary analyses examining reductions in PTSD and depression severity, and PTSD diagnostic remission, we also examined the proportion of participants who demonstrated a reliable change on the CAPS-5, PCL-5, and PHQ-9. Reliable change was calculated according to Jacobson and Truax's method of assessing meaningful change via the the Reliable Change Index (RCI) following treatment (Jacobson & Truax, 1991). The RCI is based on the standard error of measurement of the difference between individuals' baseline and posttreatment scores and is a function of the instrument's standard deviation and reliability. For the purposes of this study, we used RCI metrics based on published literature for the PCL-5, CAPS-5, and PHQ-9 (Marx et al., 2022; McMillan, Gilbody & Richards, 2010) Generalized linear models were used to evaluate group differences in the proportion of participants (with 95% confidence intervals) who had RCI at 1- and 3-month follow-up. RCI analyses only included individuals who completed baseline and the respective follow-up.

Chakraborty, H. & Gu, H. (2009). A mixed model approach for intent-to-treat analysis in longitudinal clinical trials with missing values. RTI Press Publication No. MR-0009-0903. RTI International.

Jacobson NS, Truax P. Clinical significance: a statistical approach to defining meaningful change in psychotherapy research. *J Consult Clin Psychol.* 1991: 59(1): 12-19.

Little, R. J., et al. (2012). The Prevention and Treatment of Missing Data in Clinical Trials. *New England Journal of Medicine*, 367:1355-1360.

Marx BP, Lee DJ, Norman SB, Bovin MJ, Sloan DM, Weathers FW, et al. (2022). Reliable and clinically significant change in the clinician-administered PTSD Scale for DSM-5 and PTSD Checklist for DSM-5 among male veterans. *Psychol Assess.* 34(2):197-203

McMillian D, Gilbody S, & Richards D. (2010). Defining successful treatment outcome in depression using the PHQ-9: a comparison of methods. *Journal of affective disorders*, *127*(1-3), 122–129.
