# Supplementary material for: Residential Therapy With Navigated Transcranial Magnetic Stimulation for Combat-Related PTSD: A Randomized Clinical Trial
Source: JAMA Netw Open. 2026 Apr 7;9(4):e265110. doi: 10.1001/jamanetworkopen.2026.5110 (PMC13058764; doi:10.1001/jamanetworkopen.2026.5110)
Supplement: Supplement 2. — eAppendix 1. Standard-of-Care Therapy eAppendix 2. Navigated TMS Methods eFigure 1. Robotic Stereotactic TMS Navigation System eFigure 2. PHQ-9 Outcomes by Treatment Group eTable 1. Participant Clinical Status on Study Entry eTable 2. Psychotropic Medications Alterations During Admission eTable 3. Adverse Events eTable 4. MDD Symptom Severity: Outcomes per Group by Time Point eTable 5. MDD Symptom Severity: Modeled Between-Group Differences by Study Phase [file jamanetwopen-e265110-s002.pdf]

# Supplementary Online Content

Fox PT, Salinas FS, Roache JD, et al; Consortium to Alleviate PTSD. Residential therapy with navigated transcranial magnetic stimulation for combat-related PTSD: a randomized clinical trial. *JAMA Network Open*. 2026;9(4):e265110. doi:10.1001/jamanetworkopen.2026.5110

**eAppendix 1.** Standard-of-Care Therapy

**eAppendix 2.** Navigated TMS Methods

**eFigure 1.** Robotic Stereotactic TMS Navigation System

**eFigure 2.** PHQ-9 Outcomes by Treatment Group

**eTable 1.** Participant Clinical Status on Study Entry

**eTable 2.** Psychotropic Medications Alterations During Admission

**eTable 3.** Adverse Events

**eTable 4.** MDD Symptom Severity: Outcomes per Group by Time Point

**eTable 5.** MDD Symptom Severity: Modeled Between-Group Differences by Study Phase

This supplementary material has been provided by the authors to give readers additional information about their work.

© 2026 Fox PT et al. *JAMA Network Open*.

# eAppendix 1. STANDARD-OF-CARE THERAPY

All trial participants were referred for and enrolled in the Posttraumatic Stress Disorder Intensive Inpatient Program (PTSD IIP) for combat-related PTSD offered by the Laurel Ridge Treatment Center (LRTC), San Antonio, Texas. The LRTC PTSD IIP is administered in a PTSD-specific residential unit within a military-medicine inpatient service. LRTC IIP patients are predominately ($\geq$ 90%) active-duty military personnel from all 4 service branches (Army > Air Force > Navy > Marines) with severe to extreme PTSD who have been referred by mental health providers at duty stations throughout the US and abroad. The great majority of those receiving IIP treatment return to their duty stations after program completion. The IIP average length of stay is 30 days. During this period, unit mates house together, eat together and engage in multiple activities together, both therapeutic and social.

The LRTC IIP was developed by Dr. Elna Yadin, in consultation with Dr. Edna Foa. The primary therapeutic intervention is prolonged exposure (PE), following the treatment manual of Foa, Hembree and Rothbaum[1]. The PE protocol included the four, standard, primary components: 1) imaginal exposure and processing; 2) *in vivo* exposure to trauma-related avoided situations; 3) psychoeducation about common reactions to trauma; and, 4) breathing retraining. Target traumatic events are revisited through imaginal exposures (verbal recall with audio recordings, and writing), *in vivo* exposures (daily situations causing stress), and virtual reality exposure, using virtual reality headsets of combat videos and binders of combat photographs. This is a massed-format PE, in that sessions are more often than weekly.[2]

In addition, the LRTC IIP incorporates procedural augmentations designed to maximize the benefits derived from exposure therapy informed by inhibitory learning theory.[3,4] The augmentations are organized in a manner that leverages the scheduling and group-engagement opportunities afforded by the inpatient milieu. Therapeutic augmentations included: team-based treatment; clinic-based completion of daily homework assignments; group outings for *in vivo* exposure; timeline of traumatic events creation and review; social-network engagement and education; and, team-building.

Finally, the LRTC IIP includes medication optimization via daily meetings with the admitting psychiatrist.

Brief descriptions of the required therapeutic activities follow.

Individual Prolonged Exposure. Twice weekly, individual meetings with the primary therapist, 90 minutes in duration. These focus on trauma recall and processing. Audio recordings of trauma recall are made and reviewed between sessions as homework. Done weekly with the therapist and listened to between sessions.

Goals Group Therapy. Twice daily (morning & evening), 7 days/week. One hour. Group format. Daily goals are written in the morning and reviewed in the evening.

Combat-Processing Group Therapy. Four days/week. One hour. Group format. Primary assignment and therapeutic vehicle is writing a combat timeline, as a narrative of deployment experiences and emotional reactions. These narratives are shared with peers for feedback and used to challenge maladaptive beliefs and emotions. Groups participate in re-integration outings, including stressful, potentially triggering locations as *in vivo* exposure.

Psychological Education (Psy-Ed) Group Therapy. Daily, 7 days/week. One hour. Group format. Four days per week sessions emphasize a Socratic (dialectical) approach to challenge maladaptive beliefs. Three days per week sessions emphasize the ABC (Activating Event, Belief, Consequence) cognitive therapy model.

Homework Workshop Group Therapy. Daily, 7 days/week. One hour. Group format. This entails journaling and self-assessment using the Subjective Unit Distress Scale (SUDS). Journals are reviewed in both individual and group therapy sessions.

© 2026 Fox PT et al. *JAMA Network Open*.

Social Support & Family Education. Weekly. One hour. Individual phone or Zoom family therapy and PTSD education sessions.

Yoga Group Therapy. Daily, 7 days/week. One hour. Group format. Includes: breathing exercises; meditation; mindfulness training; stress reduction and grounding skills; and, physical soothing techniques (stress balls, soothing audio recordings, etc.)

Recreation Group Therapy. Daily, 7 days/week. Two hours. Group format. Emphasizes physical activity, team building and socialization.

Art and/or Music Group Therapy. Weekly. Two hours. Group format.

Pain Treatment. Upon request. Includes massage therapy and Transcutaneous Electric Nerve Stimulation (TNS) for pain relief.

**REFERENCES.**

1. Foa E, Hembree E, Rothbaum BO. *Prolonged Exposure Therapy for PTSD: Emotional Processing of Traumatic Experiences Therapist Guide.* Oxford University Press; 2007.
2. Foa DB, McLean CP, Zang Y, et. al. (2018) Effect of prolonged exposure therapy delivered over 2 weeks vs 8 weeks vs present-centered therapy on PTSD symptom severity in military personnel: a randomized clinical trial. JAMA, 319(4):355-364.
3. Craske MG, Kiercanski, K, Selelikowsky, M, Mystikowski J, Chowdhury N, Baker A. (2008). Optimizing inhibitory learning during exposure therapy. *Behavior Research and Therapy.* 46:5-27.
4. Craske, MG, Treaner, M, Conway, CC, Sbozinek, T, and Vervilet, B (2014). Maximizing exposure therapy: An inhibitory learning approach. *Behavior Research and Therapy.* 58: 10-23.

© 2026 Fox PT et al. *JAMA Network Open*.

## eAppendix 2. NAVIGATED TMS METHODS.

TMS therapy was planned and navigated using a TMS-dedicated robotic navigation system designed and built at the Research Imaging Institute, University of Texas Health Science Center at San Antonio by Peter Fox, Jack Lancaster, Felipe Salinas and colleagues (**eFigure 1**). Planning and stereotactic navigation (coil placement) are applications of the Cortical Column Cosine (C3) model for TMS targeting (1-4). The C3 algorithm accurately predicts TMS-induced functional brain responses, both locally (5-7) and connectomically (8-11.) Personalized treatments were planned by applying treatment-planning software to per-subject structural and functional MRI. Treatments were navigated by a robotic, stereotactic coil-navigation system, algorithmically similar to that previously reported (2-4, 12), but updated for a 6-joint (6 degree-of-freedom) electromechanical arm. The positioning accuracy of this system is ~ ± 1mm (3-D location) and ± 1° (3-D orientation; roll, pitch, yaw). C3-based TMS methods and apparatus are patented (3-4, 11) and licensed for commercialization.

*Image-guided Treatment Planning.* Stimulation-site location, laterality and connectivity were informed by meta-analyses (13-14) and primary data analyses (11, 15). Stimulation targeted right anterior dorsolateral prefrontal cortex (DLPFC) by reference to standardized neuro-anatomical coordinates (MNI space), with the maximum effective electromagnetic field ($E_{eff}$; 1-4) value at an average location of (x = 38, y = 44, z = 26). Within right anterior DLPFC, coil position was adjusted per-subject to optimize local stimulation efficacy and connectivity, as follows. Using C3-based planning software, coil orientation was specified relative to cortical geometry, with the $E_{eff}$ vector perpendicular to the selected cortical-surface target, as specified by the C3 targeting model. Coil position was adjusted as needed, within limits, to allow this orientation. Cognizant that TMS recruits multi-synaptic networks determined by neural connectivity with the stimulated site, treatment locations and orientations were further adjusted per-subject to optimize functional connectivity strength with subgenual cingulate cortex (SGC) (Pearson correlation) using co-registered, resting-state fMRI. Connectivity valence was not fixed, allowing either positive or negative correlations with the seed region.

*Patient Preparation and Robotic Coil Positioning.* To receive navigated TMS, participants rested in a supine posture on a treatment table with the head restrained in a registration frame. The registration frame supported: 1) a cushioned head rest and side stabilization panels; and, 2) a mounting platform for the coordinate-measurement machine (CMM), a high-precision spatial digitizer. A digitizer was used to model skin surfaces (scalp and face) *in situ*, thereby placing the robotic arm, the TMS coil, the head, and the treatment plan in the same coordinate system.

A 6-joint robotic arm was mounted on a pedestal integrated into an equipment cart that held the image-guidance computer, the robot-arm control computer, and an isolation transformer. The 6-joint arm provided 6 degrees of freedom (i.e., 3-D location, 3-D orientation), to allow precise positioning and orientation, as specified by the image-guided treatment plan. Under technician supervision, the arm navigated the coil to the pre-selected treatment position. The robotic apparatus for stereotactic TMS navigation are illustrated in **eFigure 1**.

*Blinding.* Transcranial magnetic stimulation was administered using Cool-B65-A/P coils (MagVenture), which are double-faced coils designed for use in blinded, randomized clinical trials. One coil face is active (A), delivering intensity-calibrated TMS; the other face is passive (P), delivering sham TMS. Coil faces are unmarked, for staff and participant blinding. The coil contains an orientation sensor which prompts the operator which side to apply using the participant's randomization code. Coil-pose plans and treatment intensity settings were computed in advance of randomization, ensuring blinding to treatment arm, and delivered to the treatment site via an encrypted study portal. Randomization to treatment arm (active or sham) was performed by Data Management Core personnel on a central

© 2026 Fox PT et al. *JAMA Network Open*.

computer and transmitted to the treatment computer as an encrypted file. The randomization code determined which coil face (active or sham) was applied.

*Stimulation Parameters.* Stimulation parameters most closely resembled those of Boggio (16): intensity ~ 105% motor-threshold (MT); frequency, 20 Hz; train duration, 2 sec (40 pulses); intertrain interval, 28 sec; trains/session, 40 (1,600 pulses, total); session duration, 20 min. Low intensity was chosen to facilitate blinding by decreasing cutaneous nerve stimulation (scalp tingling) and muscle contractions. High frequency (20 Hz) was chosen to maximize administered dose (13). Unlike Boggio (16) and differing from common practice in the field, intensity was specified as $E_{eff}$ (1-7, 11-12), rather than as percent motor threshold. This approach also differs from common neuronavigational practice, which models $E_{abs}$ rather than $E_{eff}$, assuming a diffuse effect of TMS. However, MT was calculated and stimulation intensity did not exceed 105% MT.

## REFERENCES.


1. Fox PT, Narayana S, Tandon N, Sandoval H, Fox SP, Kochunov P, Lancaster JL. (2004) Column-based model of electrical field excitation of cerebral cortex. Human Brain Mapping, 22:1-16.
2. Fox PT, Narayana S, Tandon N, et al. (2006) Intensity modulation of TMS-induced cortical excitation: primary motor cortex. Human Brain Mapping, 27(6):478-487.
3. Fox PT, and Lancaster JL. (2006) US Patent 7,087,008 BS Apparatus and Methods for Delivery of Transcranial Magnetic Stimulation.
4. Fox PT, and Lancaster JL. (2010) US Patent 7,658,704 B2. Apparatus and Methods for Delivery of Transcranial Magnetic Stimulation.
5. Krieg TD, Salinas FS, Narayana S, Fox PT, Mogul DJ. (2013) PET-based confirmation of orientation sensitivity of TMS-induced cortical activation in humans. Brain Stimulation 6(6): 898-904.
6. Krieg TS, Salinas FS, Narayana S, Fox PT, Mogul DJ (2015) Computational and experimental analysis TMS-induced electric field vectors critical to neuronal activation. J Neural Eng. 12:046014.
7. Arabkheradmand F, Krieg TD, Salinas FS, Fox PT, Mogul DJ. (2019) Predicting TMS-induced activation in human neocortex using concurrent TMS/PET, finite element analysis and computational model Biomedical Physics & Engineering Express 5(2): 025028.
8. Laird AR, Robbins JM, Li K, Price LR, Cykowski MD, Narayana S, Laird RW, Franklin C, & Fox, PT. (2008) Modeling motor connectivity using TMS/PET and structural equation modeling. NeuroImage, 41(2): 424-436.
9. Narayana S, Laird AR, Tandon N, Franklin C, Lancaster JL, Fox PT. (2012) Electrophysiological and functional connectivity of the human supplementary motor area. NeuroImage. 62(1): 250-265.
10. Salinas FS, Franklin C, Narayana S, Szabo CA, Fox PT. (2106) Repetitive transcranial magnetic stimulation educes frequency-specific causal relationships in the motor network. Brain Stimulation, 9(3):406-414.
11. Fox PT, Lancaster JL, and Salinas FS. (2022) US Patent 11,458,326 B2. System, Apparatus and Methods for Image-guided, Robotically Delivered Transcranial Magnetic Stimulation (IRTMS) Treatment.
12. Lancaster JL, Narayana S, Wenzel D, Luckemeyer J, Roby J, Fox PT. (2004). Evaluation of an image-guided robotically-positioned transcranial magnetic stimulation system. Hum Brain Mapp. 22(4):329–340.


© 2026 Fox PT et al. *JAMA Network Open*.

13. Cieslik EC, Zilles K, Caspters S, Roski C, Kellermann TS, Jakobs O, Langer R, Laird AR, Fox PT, Eickhoff SB. (2012) Is There “One” DLPFC in Cognitive Action Control? Evidence for Heterogeneity From Co-Activation -Based Parcellation. Cerebral Cortex, 23:2677-1689,
14. Fox, M. D., Buckner, R. L., White, M. P., Greicius, M. D., & Pascual-Leone, A. (2012). Efficacy of transcranial magnetic stimulation targets for depression is related to intrinsic functional connectivity with the subgenual cingulate. Biological Psychiatry, 72(7):595–603.
15. Vanasse TJ, Franklin C, Salinas FS, et al.; STRONG STAR Consortium. A resting-state network comparison of combat-related PTSD with combat-exposed and civilian controls. Soc Cogn Affect Neurosci. 2019; 14(9): 933-945.
16. Boggio PS, Rocha M, Oliveira MO, et al. (2010) Noninvasive brain stimulation with high-frequency and low-intensity repetitive transcranial magnetic stimulation treatment for posttraumatic stress disorder. Journal of Clinical Psychiatry, 71(8):992-999.

© 2026 Fox PT et al. *JAMA Network Open*.

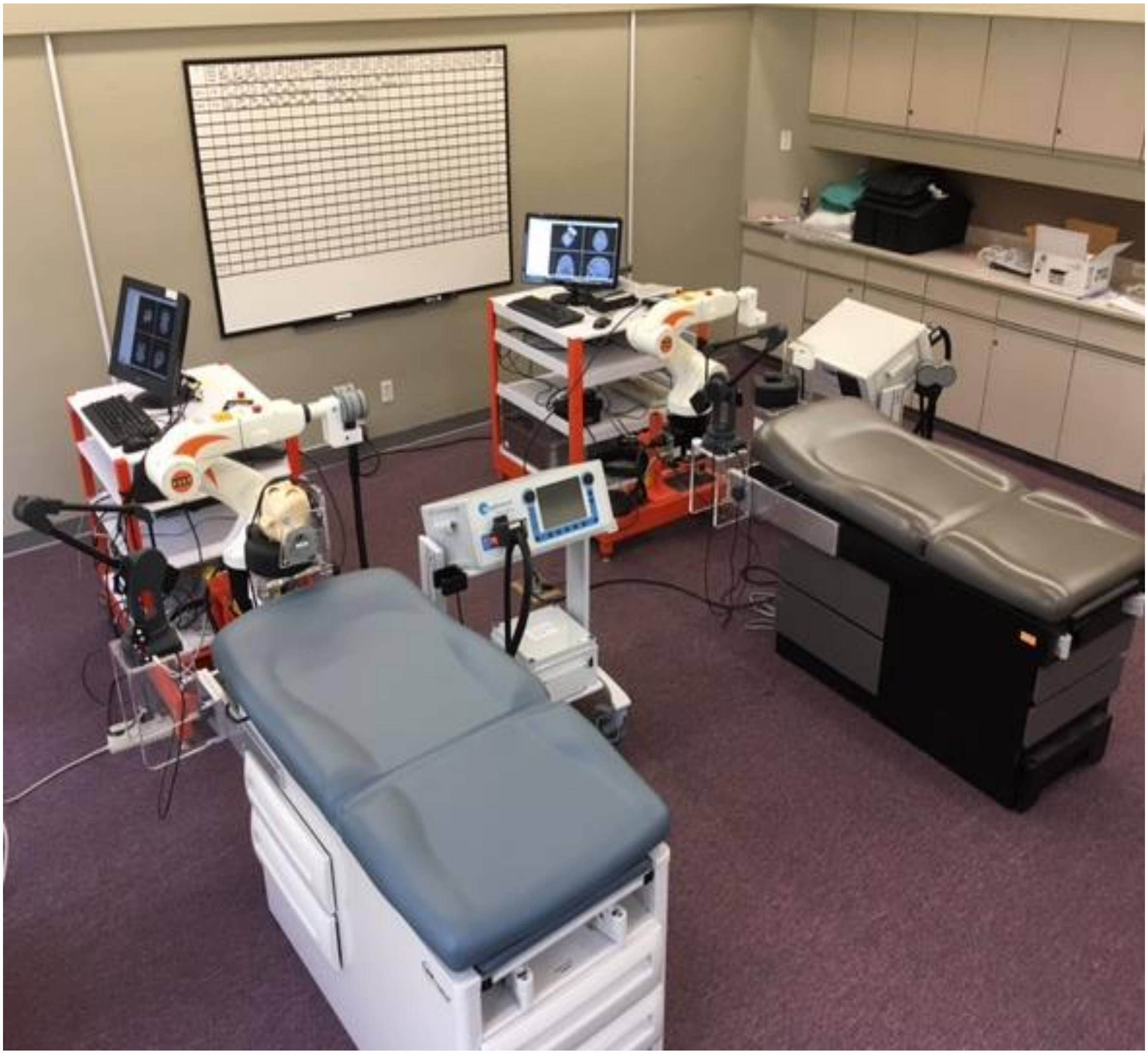

**eFIgure 1. Robotic Stereotactic TMS Navigation System.**

Illustrates image-guided, robot-navigated TMS (IR-TMS™) system in TMS Clinic. (See Navigated TMS Methods, above).

- Stereotactic navigation is controlled by computer processors on electronics cart (background). Integration by C3Nav™ software (Cerebral Magnetics, San Antonio, TX).
- Robot arm is mounted to pedestal at head of treatment table. Two-sided (active/passive) TMS coil is attached to robot tool flange by coil holder.
- Five-axis measuring arm is mounted to treatment table by registration frame at patient's right shoulder). Registration frame supports head-registration system with 2 (left/right) stabilizing panels.
- For TMS treatment, patient is supine on treatment table (foreground). Head rests on covered foam cushion. Head is restrained between acrylic stabilizing panels by gel pads over the ears and hook-and-eye strap across forehead.
- Two systems allow one technician to treat two study participants concurrently.

© 2026 Fox PT et al. *JAMA Network Open*.

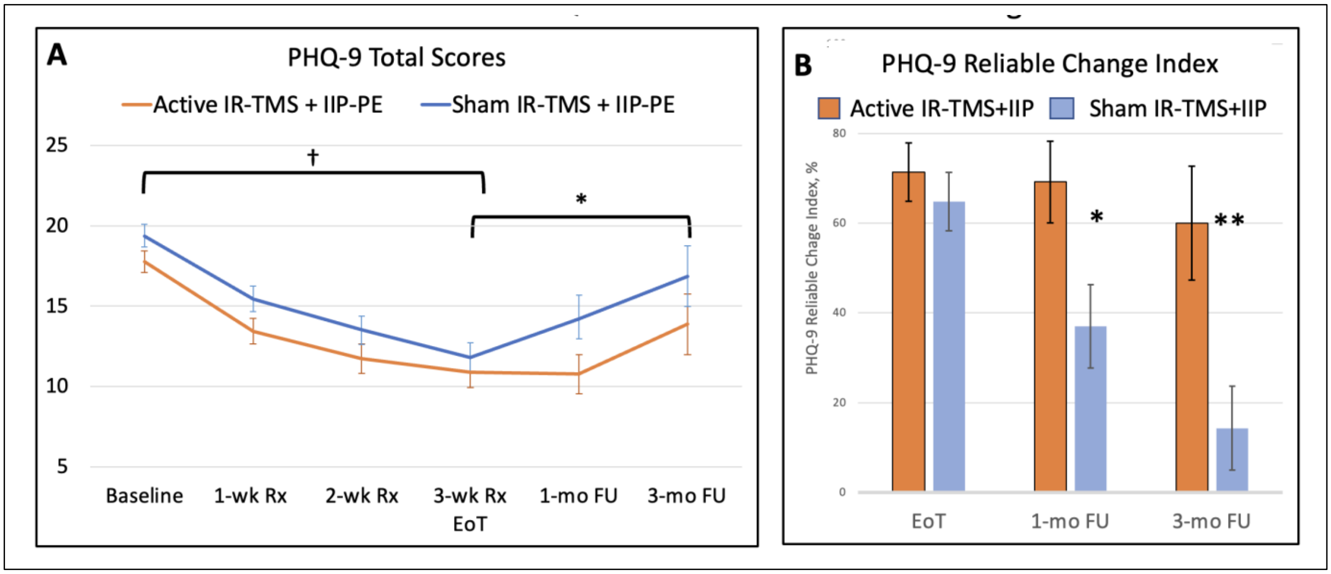


**eFigure 2. PHQ-9 Outcomes by Treatment Group**

**Panel A** plots PHQ-9 Total Score at each assessment timepoint. Scale range is from 0 (no symptoms) to 27. Within-group Treatment Phase differences from baseline, both treatment arms: †, $p < .001$. Between-group differences by study phase: *, $p < .05$.

**Panel B** plots PHQ-9 Reliable Change Index (change from baseline) at end of treatment and each follow-up timepoint. Scale range is from 0% (no patients exhibit reliable change from baseline) to 100%. Between-group differences by timepoint: *, $p < .05$; **, $p < .005$.

Error bars are +/- 1 standard error for both panels.

**Abbreviations:** EoT, end of treatment; FU, follow-up; IIP, Intensive Inpatient Program; IR-TMS, image-guided, robot-navigated transcranial magnetic stimulation; mo, month; PHQ-9, Patient Health Questionnaire 9; Rx, treatment; wk, week.

© 2026 Fox PT et al. *JAMA Network Open*.

| Participant Clinical Status at Study Entry | | |
|---|---|---|
| | **Sham+SoC** | **Active+SoC** |
| | n = 59 | n = 60 |
| PTSD diagnosis by CAPS-5[a] | 59 | 60 |
| PTSD severity by CAPS-5[b] | | |
| Total score, mean (range) | 45.9 (27-65) | 44.9 (25-66) |
| Mild | 0 | 0 |
| Moderate | 3 | 6 |
| Severe | 26 | 30 |
| Extreme | 30 | 24 |
| PTSD severity by PCL-5 | | |
| Total score, mean (range) | 60.2 (31-80) | 59.6 (35-78) |
| Depressive-symptom severity | | |
| PHQ-9, total Score, mean (range) | 19.3 (3-27) | 17.8 (4-26) |
| Anxiety symptom severity | | |
| GAD-7, total Score, mean (range) | 16.3 (4-21) | 15.8 (5-21) |
| Alcohol use | | |
| AUDIT (total score) | 5.6 (4-8) | 5.4 (4-7) |
| AUDIT-C | 2.9 (2-4) | 2.8 (2-4) |
| Mental functioning by VR-12 | 22.3 (20-25) | 24.2 (22-27) |
| Physical functioning by VR-12 | 42.5 (39-46) | 42.2 (39-46) |

**eTable 1 Participant Clinical Status on Study Entry**

**Abbreviations**: Active, active navigated transcranial magnetic stimulation; AUDIT, Alcohol Use Disorder Identification Test; CAPS-5, Clinician-Administered PTSD Scale for *DSM-5*; Generalized Anxiety Disorder-7, GAD-7; TMS, navigated transcranial magnetic stimulation; PCL-5, PTSD Checklist for *DSM-5*; PHQ-9, Patient Health Questionnaire depression module; Sham, sham navigated transcranial magnetic stimulation; SoC, standard of care residential treatment; VR-12, Veterans RAND 12-item Health Survey.

[a] Met PTSD diagnosis by CAPS-5 at baseline.

[b] Mild, ≤ 22; moderate, ≥ 23, ≤ 34; severe ≥ 35, ≤ 46; extreme, ≥ 47.

© 2026 Fox PT et al. *JAMA Network Open*.

| Psychotropic Medications Alterations During Admission | | | |
|---|---|---|---|
| | Total | Sham+SoC | Active+SoC |
| | 119 | 59 | 60 |
| **Prior Medications Discontinued** | | | |
| Antidepressants | 18 | 10 | 8 |
| Benzodiazepines | 13 | 6 | 7 |
| Other Sedative-Hypnotics | 17 | 6 | 11 |
| Anticonvulsants | 2 | 0 | 2 |
| Atypical Antipsychotics | 7 | 4 | 3 |
| Alpha/Beta Blockers | 10 | 4 | 6 |
| Other psychotropics | 9 | 5 | 4 |
| | | | |
| **Prior Medications Continued** | | | |
| Antidepressants | 98 | 49 | 49 |
| Benzodiazepines | 44 | 22 | 22 |
| Other Sedative-Hypnotics | 71 | 36 | 35 |
| Anticonvulsants | 18 | 5 | 13 |
| Atypical Antipsychotics | 23 | 12 | 11 |
| Alpha/Beta Blockers | 50 | 28 | 22 |
| Other psychotropics | 36 | 20 | 16 |
| | | | |
| **New Medications Initiated** | | | |
| Antidepressants | 62 | 36 | 26 |
| Benzodiazepines | 29 | 21 | 8 |
| Other Sedative-Hypnotics | 39 | 17 | 22 |
| Anticonvulsants | 16 | 6 | 10 |
| Atypical Antipsychotics | 14 | 7 | 7 |
| Alpha/Beta Blockers | 39 | 20 | 19 |
| Other psychotropics | 23 | 13 | 10 |

**eTable 2 Psychotropic Medications Alterations During Admission**

Psychotropic medication alterations by the attending psychiatrist during the Lead-in Phase or Treatment Phase of the RCT are enumerated.

**Abbreviations**: Active, active navigated transcranial magnetic stimulation; Sham, sham navigated transcranial magnetic stimulation; SoC, standard of care residential treatment,

© 2026 Fox PT et al. *JAMA Network Open*.

| Adverse Events[a] | | | |
|---|---|---|---|
| | Total | Sham+SoC | Active+SoC |
| | 119 | 59 | 60 |
| Headache | 34 | 19 | 15 |
| Migraine | 1 | 0 | 1 |
| Light-headedness or dizziness | 6 | 5 | 1 |
| Pain on stimulation | 3 | 0 | 3 |
| Scalp tingling or numbness | 2 | 0 | 2 |
| Muscle twitching, spasm or tension | 7 | 1 | 6 |
| Tinnitus | 2 | 2 | 0 |
| Blurred vision | 1 | 0 | 1 |
| Anxiety | 2 | 0 | 2 |
| Other discomfort | 1 | 0 | 1 |
| Foggy feeling | 1 | 1 | 0 |
| | | | |
| Total Subjects with AEs | 50 | 24 | 26 |
| Total AEs | 60 | 28 | 32 |

**eTable 3 Adverse Events**

**Abbreviations**: Active, active navigated transcranial magnetic stimulation; AE, adverse event; Sham, sham navigated transcranial magnetic stimulation; SoC, standard of care residential treatment,

[a]Each cell represents the number of AEs, not the number of subjects experiencing that AE.

© 2026 Fox PT et al. *JAMA Network Open*.

| MDD Symptom Severity: Outcomes per Group by Timepoint | | | | |
|---|---|---|---|---|
| | Score, mean (SD) n \| g[a] | | | |
| | | | Follow-up, month | |
| Outcome | Baseline | EoT | 1 | 3 |
| PHQ-9 | | | | |
| Sham + SoC | 19.34 (5.80) n = 59 \| NA, | 11.81 (6.71) n = 54 \| -1.09 | 14.19 (7.67) n = 27 \| -.60 | 16.86 (7.05) n = 14 \| -.40 |
| Active + SoC | 17.77 (5.11) n = 60 \| NA | 10.88 (6.73) n = 49 \| -1.10 | 10.77 (6.21) n = 26 \| -1.02 | 13.87 (7.31) n = 15 \| -.76 |

**eTable 4. MDD Symptom Severity: Outcomes per Group by Time Point**

**Abbreviations**: Active, active navigated transcranial magnetic stimulation; EoT, end of treatment; g, Hedges g effect size; PHQ-9, Patient Health Questionnaire, module 9; SD, standard deviation; Sham, sham navigated transcranial magnetic stimulation; SoC, standard of care residential treatment,

[a] Hedges' g calculated within-arm, comparing within-arm scores at endpoint of interest to within-arm baseline scores.

© 2026 Fox PT et al. *JAMA Network Open*.

| MDD Symptom Severity: Modeled Between-Group Differences by Study Phase[a] | | | | | | | | |
|---|---|---|---|---|---|---|---|---|
| Treatment Phase | | | | | Follow-up Phase | | | |
| MD [95% CI][b] | T[b] | P value[b] | g[c] | | MD [95% CI][b] | T[b] | P value[b] | g[c] |
| PHQ-9 | | | | | | | | |
| -1.58 [-3.75, .58] | -1.43 | .078 | -.14 | | -3.45 [-6.86, -.03] | -1.98 | .025 | -.41 |

**eTable 5. MDD Symptom Severity: Modeled Between-Group Differences by Study Phase**

Compares Active TMS to Sham TMS by study phase. Negative mean difference indicates greater symptom remediation by Active TMS.

**Abbreviations**: CI, confidence interval; g, Hedges g effect size; MD, between-group modeled difference in change over study phase; PHQ-9, Patient Health Questionnaire, module 9; T, t-statistic.

[a] Both treatment arms demonstrated significant MDD symptom reductions ($p < 0.001$) on both outcome measures during Treatment Phase (baseline to end of treatment).

[b] Modeled mean difference (active group intercept – sham group intercept), 95% CI, T-statistic and p-value at conclusion of phase.

[c] Hedges' g calculated based on descriptive statistics between arms at the time point of interest.

© 2026 Fox PT et al. *JAMA Network Open*.
