# Supplementary material for: Residential Therapy With Navigated Transcranial Magnetic Stimulation for Combat-Related PTSD: A Randomized Clinical Trial
Source: JAMA Netw Open. 2026 Apr 7;9(4):e265110. doi: 10.1001/jamanetworkopen.2026.5110 (PMC13058764; doi:10.1001/jamanetworkopen.2026.5110)
Supplement: Supplement 3. — Data Sharing Statement [file jamanetwopen-e265110-s003.pdf]

Data Sharing Statement

Fox. Residential Therapy With Navigated Transcranial Magnetic Stimulation for Combat-Related PTSD. *JAMA Netw Open*. Published April 07, 2026.
doi:10.1001/jamanetworkopen.2026.5110

Data
Additional Information: Clinical Trails.gov; https://clinicaltrials.gov; Identifier NCT02853032
Data available: Yes
Data types: Deidentified participant data
How to access data: repository@strongstar.org
When available: With publication

Supporting Documents
Document types: Informed consent form
How to access documents: repository@strongstar.org
When available: With publication

Additional Information
Who can access the data: Researchers whose proposed use of the data has been approved
Types of analyses: For a specified purpose
Mechanisms of data availability: With investigator support, after approval of a proposal, and with a signed data access agreement
Any additional restrictions: Agreement to not attempt to identify or contact research participants.
